# Exercise Training Stimulates the Release of Glutathione Peroxidase 1 (GPX1)‐Enriched Extracellular Vesicles That Promote Angiogenesis

**DOI:** 10.1096/fj.202505096RR

**Published:** 2026-06-18

**Authors:** Alexander M. Fliflet, Ray A. Spradlin, Yanqi Tan, Ane Nishitha Vijayan, Sung Jun Choi, Wei‐Chun Kao, Edita Aksamitiene, Michael Nelappana, Shengzhe Ding, Kai‐Yu Huang, Aryaman Joshi, Nurila Kambar, Tom Bludgen, Madeleine Meehan, Jayna L. Boss, Michael Knipp, Hongming Fan, Cecilia Leal, Roger A. Sunde, Lawrence W. Dobrucki, Stephen A. Boppart, Hyun Joon Kong, Jonathan V. Sweedler, Marni D. Boppart

**Affiliations:** ^1^ Department of Health and Kinesiology University of Illinois Urbana‐Champaign Urbana Illinois USA; ^2^ Beckman Institute for Advanced Science and Technology University of Illinois Urbana‐Champaign Urbana Illinois USA; ^3^ Department of Chemistry University of Illinois Urbana‐Champaign Urbana Illinois USA; ^4^ Department of Bioengineering University of Illinois Urbana‐Champaign Urbana Illinois USA; ^5^ Department of Chemical and Biomolecular Engineering University of Illinois Urbana‐Champaign Urbana Illinois USA; ^6^ Department of Materials Science and Engineering University of Illinois Urbana‐Champaign Urbana Illinois USA; ^7^ Department of Nutritional Sciences University of Wisconsin‐Madison Madison Wisconsin USA; ^8^ Department of Electrical and Computer Engineering University of Illinois Urbana‐Champaign Urbana Illinois USA; ^9^ NIH/NIBIB Center for Label‐Free Imaging and Multiscale Biophotonics (CLIMB), university of Illinois Urbana‐Champaign Urbana Illinois USA; ^10^ Department of Cell and Developmental Biology University of Illinois Urbana‐Champaign Urbana Illinois USA

**Keywords:** angiogenesis, antioxidants, exercise, extracellular vesicles, glutathione peroxidase 1

## Abstract

An acute bout of high intensity exercise can transiently increase circulating extracellular vesicles (EVs) that possess beneficial molecular cargo. However, no studies to date have comprehensively evaluated plasma quantity, protein content, and function of EVs collected from blood after multiple bouts of endurance exercise. Here we demonstrate that 4 weeks of voluntary wheel running increases plasma EV quantity when collected immediately after the last bout of training in mice. These EVs (ExerVs) are enriched in oxidoreductases, including the antioxidant glutathione peroxidase 1 (GPX1). Repeated, systemic injections of ExerVs into sedentary recipient mice twice per week for 4 weeks did not alter mitochondrial content or function, fiber size, or fiber type, but increased capillary density and perfusion in skeletal muscle. ExerVs also stimulated tube formation and branch lengthening in vitro and improved the recovery of capillary content after a period of disuse in vivo. ExerVs isolated from GPX1^−/−^ mice lacked the ability to stimulate vessel formation, whereas GPX1‐encapsulated liposomes robustly increased capillary growth, both in vitro and in vivo. The results from this study suggest that circulating ExerVs positively impact vascular structure and function in skeletal muscle in a manner that may be dependent on GPX1.

## Graphical abstract

Exercise Training Stimulates the Release of Glutathione Peroxidase 1 (GPX1)‐Enriched Extracellular Vesicles that Promote Angiogenesis. The results from this study suggest that EVs isolated from blood immediately after the last bout of endurance exercise training (ExerV) are enriched in antioxidants. ExerVs stimulate vascular growth both in vitro and in vivo in a manner that may be dependent on the presence of GPX1 based on experiments conducted with GPX1−/− ExerVs and liposomes enriched in GPX1.

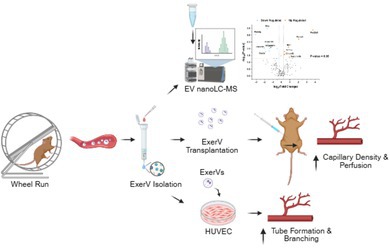

## 1 Introduction

Repeated bouts of endurance exercise can promote the metabolic health of skeletal muscle while simultaneously improving function and preventing disease in multiple organ systems. Despite the well‐known beneficial effects of endurance exercise training, the precise mechanisms driving systemic adaptations as a result of tissue crosstalk remain unclear. Studies suggest that a wide variety of cells release humoral factors or exerkines into circulation during exercise which contribute to whole‐body health via intercellular communication across organs ([1, 2, 3, 4, 5], [6, 7, 8, 9, 10]). For example, a recently developed cell type‐specific secretome map suggests that Pdgfrα^+^ cells (fibroblasts, mesenchymal stem cells, progenitor cells) resident in most tissues are exquisitely sensitive to exercise in mice and secrete factors into blood that are necessary for adaptation [11]. Mapping the complex connectome of circulating factors that are responsible for maintenance of health and subsequently using this tool as a predictive assessment to inform healthy behaviors is a priority. However, fundamental questions remain regarding the mechanisms by which exercise facilitates the release of exerkines from cells and the primary means of transport within the bloodstream.

The classic secretory pathway is the most well understood mechanism for release of proteins from cells, which includes trafficking of nascent proteins to the endoplasmic reticulum and Golgi apparatus for modification, packaging within a vesicle, and fusion of the vesicle with the cell surface for secretion. More recently, it has been recognized that cells also secrete molecular cargo (proteins, nucleic acids, lipids) via simple budding and fission at the membrane (microvesicles, large extracellular vesicles (EVs), > 200 nm) or via a more complex endosomal sorting complex required for transport (ESCRT) pathway (exosomes, small EVs, 30–200 nm) [12]. Despite growing evidence that an acute bout of high intensity exercise can stimulate the release of small, lipid‐bound EVs into blood (herein denoted as “ExerVs”) ([13, 14, 15, 16, 17, 18, 19, 20, 21]), minimal information exists regarding the impact of endurance exercise training on ExerV quantity, particularly when isolated immediately after the last bout of exercise. Additionally, ExerVs released after an acute bout of exercise appear to possess beneficial health‐promoting molecular cargo, such as antioxidants [13, 17, 19, 20, 22], yet parallel studies are lacking with regard to training. Finally, the function of ExerVs released acutely after training has not been fully tested in vitro and in vivo. One study has demonstrated a protective effect of ExerVs collected after 3 weeks of swim training in mice on cardiac muscle cells following ischemic injury [14], and another mouse study has reported that ExerVs collected after only 2 weeks of voluntary wheel running can induce capillary tube formation in vitro in a manner dependent on superoxide dismutase 3 (SOD3) [23]. However, neither of these studies have simultaneously and comprehensively evaluated plasma quantity, protein content, and function of ExerVs released acutely after training.

The goal of this study was to examine the impact of 4 weeks of voluntary wheel running on EV quantity and protein content by nano liquid chromatography‐mass spectrometry (nanoLC‐MS), then determine the capacity for systemically injected ExerVs to induce beneficial metabolic and structural adaptations in sedentary mice, specifically in skeletal muscle. We hypothesized that endurance training would elicit an increase in circulating ExerVs when assessed immediately after the last bout of exercise and that these ExerVs would be enriched in beneficial exerkines that phenocopy the effects of exercise. Here we demonstrate that plasma ExerV quantity is significantly increased after the final bout of exercise following 4 weeks of endurance training, and that these ExerVs are enriched in oxidoreductases, including the antioxidant glutathione peroxidase 1 (GPX1). ExerVs systemically administered to sedentary animals did not induce mitochondrial biogenesis or function in skeletal muscle, but increased capillary density and blood perfusion. ExerVs also increased endothelial cell tube formation in vitro and recovered capillary content in skeletal muscle after disuse in vivo. GPX1^−/−^ ExerVs did not elicit these beneficial effects, whereas GPX1‐encapsulated liposomes robustly increased tube formation and increased capillary recovery post‐disuse. Overall, this study is the first to demonstrate an important role for ExerVs collected after training to positively impact angiogenesis in a manner that may be dependent on GPX1.

## 2 Materials and Methods

### 2.1 Animals

Protocols for animal use were approved by the Institutional Animal Care and Use Committee (IACUC) of the University of Illinois at Urbana‐Champaign (#22061, #22062, #24063). Young adult (4‐month‐old) C57BL6/J (Wild type, WT) male mice were ordered from Charles River Laboratories (Wilmington, MA, USA) or Jackson Laboratory (Jackson, ME, USA). GPX1^−/−^ male mice (4–6‐month‐old) were obtained from Dr. Roger Sunde at the University of Wisconsin‐Madison [24]. R26‐mTmG mice were ordered from Jackson Laboratory (#007676) and plasma EVs from these mice were used to verify their ability to localize to skeletal muscle after injection. Animals were housed under a 12 h light/dark cycle (lights on 07:00 to 19:00 h) in a pathogen free, temperature‐controlled facility and given *ad libitum* access to food, standard laboratory chow, and water.

### 2.2 Endurance Exercise Training

Mice (WT; GPX1^−/−^) received horizontal running wheels in cages, either locked (sedentary controls) or unlocked (Low‐Profile Wireless Running Wheel, Med Associates Inc., Fairfax, VT, USA). Distance was automatically logged for 28 days. On the last night of wheel running, food was removed from cages at ~7 PM, then mice were euthanized immediately after running ended (~4 AM). Body and muscle weights were recorded, and blood was collected in EDTA‐coated syringes via cardiac puncture.

### 2.3 EV Isolation and Characterization

EV isolation, characterization, and injection methods were carried out according to the MISEV guidelines [25] and were consistent with those described by Wu et al. [26]. Blood samples were centrifuged at 4000 g for 15 min to obtain platelet‐free plasma. EVs were isolated via size exclusion chromatography (SEC) (Fractions #4–6 using qEV single or Fractions #7–10 using qEV original; Izon Science, Medford, MA, USA) and ultrafiltration (Amicon Ultra‐2 Centrifugal Filter Unit, 30 kDa cutoff; Millipore, St. Louis, MO, USA). The protein fractions (#8–10 qEV single or #16–25 qEV original) were also collected and used as an EV‐depleted control in some experiments. EVs were aliquoted (3 × 10^8^ particles/injection), temporarily stored at −80°C, thawed on ice, and used within 30 days. EV concentration and size were evaluated using nanoparticle tracking analysis (NTA; NanoSight NS300, Malvern Panalytical, Malvern, United Kingdom). Protein concentration was assessed by bicinchoninic acid (BCA) assay (Pierce, Thermo Fisher Scientific, Grand Island, NY, USA).

### 2.4 Cryogenic Transmission Electron Microscopy (Cryo‐EM)

4 μL of isolated EV solution was applied to glow‐discharged QUANTIFOIL R 2/2 copper mesh grids (Electron Microscopy Sciences) using a Vitrobot Mark IV (Thermo Fisher Scientific) under controlled conditions of 24°C and 100% relative humidity. Grids were blotted for 6 s with a blot force of 1 and subsequently vitrified in liquid ethane. Imaging was performed using a 200 keV Glacios transmission electron microscope (Thermo Fisher Scientific) equipped with a Falcon 4 direct electron detector. Images were acquired using EPU automated acquisition software (Thermo Fisher Scientific).

### 2.5 EV Protein Extraction, Digestion, and Clean‐Up

~50 μL of each EV sample was lysed using the Perfect‐FOCUS kit (G‐Biosciences, St. Louis, MO) following the manufacturer's instructions. After the proteins were extracted, the samples were dried in a SpeedVac (Genevac, Ipswich, Suffold, UK), then reconstituted in 100 μL of 6 M urea/50 mM Tris–HCl (pH 8), followed by the addition of 5 μL of 200 mM dithiothreitol (DTT)/50 mM Tris–HCl (pH 8). Samples were incubated for 1 h at RT (~20°C) prior to adding 20 μL of 200 mM iodoacetamide/50 mM Tris–HCl (pH 8) to each sample. After an additional 1 h incubation at RT in the dark, 20 μL of 200 mM DTT/50 mM Tris–HCl (pH 8) was added to the samples to neutralize unreacted iodoacetamide. After the final 1‐h incubation at RT in the dark, each sample was diluted with 775 μL of 1 mM CaCl_2_/50 mM Tris–HCl (pH 7.6). MS Grade Modified Trypsin Protease (Thermo Fisher Scientific) was added to each sample at a trypsin: protein ratio of 1:20 (w/w). The samples were digested for 16 h in a 37°C water bath. The enzymatic reaction was quenched by adding 1 μL of formic acid (FA) to reduce the pH to 3–4, and then the samples were dried down completely in the SpeedVac. Next, the dried protein digests were reconstituted in 200 μL of 0.1% formic acid (FA) in water, and they were desalted using Pierce Peptide Desalting Spin Columns (ThermoFisher Scientific) as per the manufacturer's instructions. The tryptic peptides were eluted from the spin columns with 600 μL of 50/49.9/0.1 acetonitrile/water/FA, and then the eluate was dried down completely in the SpeedVac and stored at −80°C until further use.

### 2.6 Liquid Chromatography‐Trapped Ion Mobility Spectrometry‐Mass Spectrometry Analysis

Samples were reconstituted in 20 μL of 0.1% FA in water, and peptide concentrations were determined using Pierce Quantitative Fluorescence Peptide Assay (Thermo Fisher Scientific) according to the manufacturer's instructions. LC separation of the tryptic peptides was performed on a nanoElute LC system (Bruker Daltonics) with a pre‐concentration setup. Mobile phases consisted of LC–MS grade water containing 0.1% FA (solvent A) and LC–MS grade acetonitrile containing 0.1% FA (solvent B). For each sample analysis, 200 ng of the peptide digest were loaded on Acclaim PepMap 100 C18 (Thermo Scientific, 0.3 mm x 5 mm, 100 Å pore size) using solvent A, for approximately 5 min, depending on the backpressure generated by the sample. The peptides were then separated on a nanoElute FIFTEEN column (Bruker Daltonics, 75 μm × 150 mm, C18, 1.9 μm particles, 120 Å pore size). The separation was carried out at 40°C with a flow rate of 400 nL/min using the following gradient: 2%–10% B from 0 to 2 min, 10%–35% B from 2 to 90 min, 35%–45% B from 90 to 105 min, followed by wash and equilibration steps during which data was not acquired. The LC was coupled to a timsTOF Pro mass spectrometer MS (Bruker Daltonics) with a CaptiveSpray ion source. The MS was operated in parallel accumulation‐serial fragmentation (PASEF) mode with TIMS on and dynamic exclusion, 10 PASEF MS/MS scans per 1.1 s cycle. Active exclusion for precursor ions was selected to release after 0.40 min, and precursors were reconsidered for analysis if the current intensity/previous intensity was greater than 4.0. The mass range was set between 100 and 1700 *m/z*, and the ion mobility range was set between 0.60 and 1.60 V·s/cm^2^ with a ramp time of 100.0 ms. The intensity threshold was set to 5000 units and the target intensity was set to 20 000 units. The collision energy was ramped between 20.00 to 59.00 eV as a function of ion mobility.

### 2.7 Database Search and Label‐Free Quantitation

The acquired raw data from each LC–MS analysis was imported onto PEAKS Online X (Bioinformatics Solutions Inc., Waterloo, ON) for peptide sequencing and protein identification. The protein database for mouse species ( *Mus musculus* ) containing 17 058 reviewed proteins was downloaded from UniProt. The following parameters were employed for *de novo* peptide sequencing and protein identification via database search: precursor mass error tolerance = 15 ppm, fragment mass error tolerance = 0.05 Da, enzyme = trypsin, digest mode = specific, peptide length was set between 6 and 45 residues, and the maximum number of missed cleavages was set to 3. Carbamidomethylation of cysteine residues was set as a fixed modification and oxidation of methionine residues was set as a variable modification. Search result filters were set to a peptide false discovery rate (FDR) of 1% and a protein filter was set to a −10log *p* ≥ 20. For label‐free quantitation, the PEAKS Q module was employed to extract the peak areas of the peptides in each sample. The following parameters for the PEAKS Q quantitation were as follows: mass error tolerance = 15 ppm, retention time shift tolerance was set to “auto detect”, CCS error tolerance = 0.05, and feature intensity ≥ 0. The following peptide filters were selected: average area ≥ 0, peptide ID count ≥ 1, charge between 1 and 10, have at least 2 confident samples per group, and use in group coefficient of variation filter. No normalization was applied, since normalization was performed elsewhere, as described in the next section. Peptide ID transfer was enabled to reduce the number of missing peptides across samples.

### 2.8 Proteomic Evaluation Statistical Analysis

Peptide peak areas obtained from PEAKS Q were imported onto the Normalyzer (version 1.14.0) online tool for normalization, whereby peak areas were log_2_ transformed and then normalized by variance stabilization normalization (VSN). This method of normalization has been shown to be well‐suited for bottom‐up proteomics in terms of minimizing random intragroup variation across samples during the MS acquisition for accurate quantitation. After removal of immunoglobulins and keratins, Student's unpaired *t*‐tests were performed between the two sample groups to obtain *p*‐values, and a multiple hypothesis testing was corrected using the Benjamini‐Hochberg FDR method to obtain Q‐values. Protein classification was conducted using the PANTHER Classification System using *Mus musculus* genes as the reference.

### 2.9 Systemic EV Injections Into Sedentary Mice (Adaptation Study)

Young adult (4‐month‐old) C57BL/6J male mice received the following treatments: (1) PBS as a vehicle control, (2) EVs derived from sedentary mice (SedV) as a condition control, and (3) EVs derived from exercised mice (ExerVs). Based on prior results [26], EVs (3 × 10^8^ per injection; pooled from donor mice) were intraperitoneally injected in a total volume of 100 μL PBS twice per week for 4 weeks. Mice were euthanized by CO_2_ asphyxiation 24 h after the last injection, and body and gastrocnemius muscle weights were recorded.

### 2.10 Intramuscular EV Injection Into Immobilized Mice (Disuse Recovery Study)

Young adult (4‐month‐old) C57BL/6J male mice were anesthetized using isoflurane (5% induction, 2.5% maintenance) and subjected to unilateral limb immobilization using an Autosuture Royal 35 W skin stapler (eSutures, Mokena, IL, USA), as previously described [26, 27, 28]. Buprenorphine (2.5 μg/g of body weight; Ethiqa XR, Fidelis Animal Health, North Brunswick, NJ, USA) was administered before the surgical procedure to relieve pain. Mice were monitored for up to 5 days following the procedure and were administered another dose of buprenorphine if pain persisted. Staples were removed after 14 days and mice were allowed free movement within the cage for an additional 3 days. Immediately prior to remobilization, mice received a single intramuscular (i.m.) injection of either: (1) PBS as a vehicle control, (2) protein‐enriched EV‐depleted plasma as an additional control (Fractions #8–10 from SEC), (3) SedVs, (4) ExerVs, (5) SedVs from GPX1^−/−^ mice, or (6) ExerVs from GPX1^−/−^ mice. Based on prior results [26], 5 × 10^8^ EVs were directly injected into the tibialis anterior (TA) in a total volume of 30 μL PBS. For the EV‐depleted plasma, protein concentrations were assessed by BCA assay and 20 μg protein was injected in a total volume of 30 μL PBS.

### 2.11 GPX1‐Encapsulated Liposome Assembly and Injection Into Immobilized Mice (Disuse Recovery Study)

2.5 mg of 1,2‐dipalmitoyl‐sn‐glycero‐3‐phosphocholine (DPPC) (Avanti Polar Lipids, US) was dissolved in 0.25 mL of chloroform and subjected to rotary evaporation under vacuum to form a thin lipid film. The lipid film was hydrated with 2.5 mL of PBS containing either 0.5 μg of GPX1 and 5 mg of hyaluronic acid‐graft‐octadecylamine (HA‐g‐C18) [29] or 5 mg of HA‐g‐C_18_ alone in PBS. The resulting mixture was then vortexed for 1 min and extruded through a 0.2 μm polycarbonate membrane (Whatman, US) for 21 times while maintained at 45°C to obtain uniform liposomes (LPs). The LPs were collected by centrifugation at 14000 × g for 15 min at 4°C. After assembling the GPX1 LPs, young adult mice (4‐month‐old) C57BL/6J male mice were immobilized using the same procedures as above. Staples were removed after 14 days, and mice were allowed free movement within the cage for 3 days. Immediately prior to remobilization, each mouse received a single TA muscle injection of either empty LPs or LPs loaded with GPX1 protein (GPX1) suspended in 30 μL PBS.

### 2.12 Localization of Injected EVs by Multimodal Microscopy

EVs were isolated from the plasma of sedentary mTmG mice and injected intraperitoneally (3 × 10^8^) into WT mice. Skeletal muscle was dissected 24 h later and tdTomato^+^ EVs were detected in the collagen‐enriched interstitium by multimodal multiphoton imaging [30]. Briefly, multiphoton imaging was performed on a custom‐built Simultaneous Label‐free Autofluorescence Multi‐harmonic (SLAM) microscope. A 1030 nm femtosecond laser operating at a repetition rate of 10 MHz served as the excitation source. The laser output was coupled into a photonic crystal fiber (PCF) to generate a supercontinuum covering approximately 950–1150 nm, which was used as the excitation beam for all experiments. The broadband excitation was collimated, routed through the scanning optics, and focused onto the sample by a high numerical aperture objective. The emitted nonlinear signals were collected and directed to a multichannel detection module, where the emission was spectrally separated into four parallel detection arms nominally assigned to NADH, FAD, SHG, and THG. Each channel was defined by an appropriate bandpass filter and detected by an individual photomultiplier tube. For imaging tdTomato‐labeled extracellular vesicles (EVs) in mouse skeletal muscle ex vivo, the detection path normally configured for FAD autofluorescence was modified by replacing the FAD bandpass filter with a bandpass filter matched to the tdTomato emission bandwidth (593 nm with a bandwidth of ±20 nm; detection window = 573–613 nm), enabling selective detection of tdTomato fluorescence while leaving the remaining channels (NADH, SHG, and THG) unchanged. Images of mouse skeletal muscle tissue were acquired by raster scanning over a 400 μm × 400 μm field of view. For the representative image shown, only the SHG channel (collagen fibers) and the tdTomato channel (EVs) are displayed.

### 2.13 Evaluation of Skeletal Muscle Blood Perfusion

Laser Speckle Contrast Imaging (LSCI) is a non‐invasive optical imaging technique widely employed for assessing blood flow dynamics in preclinical and clinical research. In this study, we utilized the MOORFLPI‐2 system (Moor Instruments, UK) to monitor vascular perfusion in murine models. The MOORFLPI‐2 system offers high‐resolution, real‐time imaging capabilities, making it an ideal tool for evaluating microcirculatory changes in small animals. Briefly, mice were anesthetized and positioned on a heated platform to maintain physiological body temperature. The region of interest (paw) was exposed and illuminated with the system's laser source. Blood flow images were captured under controlled conditions, and the data were processed using the MOORFLPI‐2 software suite by segmenting regions of interest and quantifying perfusion in individual limbs. The software's advanced analysis tools facilitated the extraction of spatial and temporal perfusion metrics, which were subsequently used to evaluate vascular responses to experimental interventions.

### 2.14 Immunofluorescence of Tissue Sections

For assessment of fiber size and type, skeletal muscles (gastrocnemius for Adaptation Study; TA for Disuse Recovery Studies) were immediately frozen in liquid nitrogen‐cooled isopentane after dissection and 10 μm sections were obtained using a CM3050S cryostat (Leica Biosystems, Wetzlar, Germany). Sections were fixed in ice‐cold acetone, washed with PBS, and incubated with primary antibodies. To visualize the outline of the muscle membrane, sections were incubated with rabbit anti‐mouse dystrophin (ab15277, 1:200; Abcam, Cambridge, MA, USA). The following fiber‐type specific antibodies were used to distinguish different fiber types: IgG1 anti‐Type IIa MHC (SC‐71, 1:50; Developmental Studies Hybridoma Bank (DSHB), University of Iowa, Iowa City, IA, USA), mouse IgM anti‐Type IIb MHC (BF‐F3, 1:50; DSHB), and IgG2b anti‐Type 1 (BA‐D5, 1:50, DSHB). To visualize capillaries, sections were incubated with rat anti‐mouse CD31 (clone 390, 1:100, No. 14–0311‐85; Thermo Fisher Scientific). For assessment of collagen, sections were incubated in rabbit anti‐mouse collagen type 1 antibody (ab34710, 1:100; Abcam, Cambridge, MA, USA). For quantitation of size, 10× images were randomly obtained throughout the muscle section, and the CSA of approximately 1500 fibers was analyzed using SMASH software. For capillary analyses, five 20× images were captured, and the number of CD31‐positive capillaries was manually counted. Capillaries were normalized to the tissue area (density, #/mm^2^) and/or the number of fibers (C:F, #/#). For quantitation of collagen, ten 20× images were obtained, and the percentage of collagen‐positive area was assessed using ImageJ software. All images were obtained using a Zeiss Axiocam (Zeiss, Thornwood, NY, USA) digital camera and ZEN software. All investigators were blinded to sample information during the analyses.

### 2.15 Assessment of Mitochondrial Content

Mitochondrial content was assessed via qPCR and citrate synthase (CS) activity assay. Methods to analyze mitochondrial DNA (mtDNA) to nuclear DNA (nDNA) ratio in mouse tissues was outlined by Quiros et al. [31]. Briefly, 30 mg of gastrocnemius muscles were lysed in ice‐cold lysis buffer (LB; 100 mM NaCl, 10 mM EDTA, 0.5% sodium dodecyl sulfate (SDS), 20 mM Tris–HCl, and double‐distilled (dd) H_2_O). Samples were incubated with Proteinase K (Roche, Millipore Sigma, St. Louis, MO, USA) at 55°C overnight. Samples were incubated with RNase A (Thermo Fisher Scientific, Grand Island, NY, USA) at 37°C for 30 min. Samples were centrifuged at 15000 × g at 4°C for 10 min with 7.5 M ammonium acetate and isopropanol. The pellet pellet was resuspended in TE buffer (10 mM Tris‐Cl, 1 mM EDTA, and ddH_2_O). Sample DNAs were diluted to a final concentration of 10 ng DNA/μL. Subsequently, 2 μL of each sample was used as a template in a qPCR assay using the QuantStudio 7 Pro real‐time PCR (Thermo Fisher Scientific) with TaqMan Universal PCR Master Mix (Thermo Fisher Scientific). *Nd1* (Mm04225274_s1; Thermo Fisher Scientific) and *Actl9* (Mm00809079_s1; Thermo Fisher Scientific) were selected to quantify mtDNA and nDNA, respectively. The ratio of mtDNA/nDNA was calculated using ΔΔC_t_ values to calculate fold changes. To assess CS activity, gastrocnemius muscles were lysed and homogenized using a solution of 10× RIPA, phosphatase inhibitor (PhosSTOP; Roche, Sigma‐Aldrich Inc., St. Louis, MO, USA), protease inhibitor (Complete Mini; Rosche. Sigma‐Aldrich Inc.) and PMSF. Protein quantification was assessed via BCA assay (Pierce, Thermo Fisher Scientific). Muscle homogenates were analyzed by MitoCheck Citrate Synthase Activity Assay kit (No. 701040; Cayman Chemical, Ann Arbor, MI, USA) according to the manufacturer's protocol. Raw CS activity values were normalized to protein concentration values.

### 2.16 Impact of EVs on HUVEC Viability In Vitro

Human Umbilical Vein Endothelial Cells (HUVEC, Lonza #CC‐2519A; Cambridge, MA, USA) were cultured on 96‐well polystyrene plates (1 × 10^4^ cells/well) and incubated with Endothelial Cell Basal Medium (EBM‐2; Lonza), supplemented with 1% penicillin–streptomycin (PS; Thermo Fisher Scientific), and 2% exosome‐free fetal bovine serum (FBS; Thermo Fisher Scientific) until 80% confluent. Media or EVs (4 × 10^7^ particles, *n* = 5/group) were added in triplicate, and cell viability was assessed at 24 h via MTT assay following manufacturer's instructions (M6494, Thermo Fisher Scientific).

### 2.17 Impact of EVs on HUVEC Tube Formation In Vitro

96‐well polystyrene plates were coated with Matrigel (Matrigel Basement Membrane Matrix, Phenol Red‐free, LDEV‐free; Corning, Tewksbury, MA, USA) and allowed to solidify for 30 min at 37°C. HUVECs (2 × 10^4^ cells/well, Lonza #CC‐2519A) were cultured in Matrigel‐coated 96‐well polystyrene plates as described above. Each plate was treated with either media or EVs (4 × 10^7^, *n* = 5/group) in triplicate. After 8 h, a single bright‐field microscopy image of forming tubes was obtained in the center of the well using 5× or 10× objectives. Images were analyzed via Angiogenesis Analyzer Plugin via ImageJ.

### 2.18 Validation of GPX1 Protein in EVs

GPX1 protein was detected by Multistrip Western Blotting as described previously [32, 33] with slight modifications. Briefly, 100 μL of each isolated EV suspension was lysed in 100 μL ice‐cold lysis buffer (50 mM HEPES (pH 7.4), 150 mM NaCl, 1% Triton X‐100, 1 mM EGTA, 10% Glycerol, 0.5% sodium deoxycholate and 0.1% SDS) supplemented with protease (#P‐1512; A.G. Scientific, San Diego, CA) and phosphatase inhibitor (Millipore‐Sigma) cocktails. Each lysate was then mixed with 75 μL 4× NuPAGE LDS Sample Buffer and 30 μL 0.5 M DTT, boiled at 80°C for 10 min and cooled down to RT. Following equal sample loading (28 μL/lane) and LDS‐PAGE at 130 V under reducing conditions, each 10‐well 1.0 mm NuPAGE 4%–12% gradient Bis‐Tris gel (Thermo Fisher Scientific) was subdivided into three horizontal strips, containing a migratory region for 250–75 kDa, 75–37 kDa and 37–15 kDa molecular weight (MW) proteins, as indicated by the migration of Precision Plus Protein All Blue Prestained Protein Standards (Bio‐Rad, Hercules, CA) ladder loaded onto lanes #1 and #10 at 10 μL/lane. After protein transfer at 30 V for 90 min onto 0.2 μm pore‐size nitrocellulose membranes (Bio‐Rad), and 1 h incubation in 3% bovine serum albumin (BSA) solution, the corresponding membranes were probed with rabbit polyclonal anti‐ALIX (#M01751; BosterBio, Pleasanton, CA), and mouse monoclonal anti‐GPX‐1 (8B10) Picoband (#M01019‐2; BosterBio) antibodies at a working 0.5–1 μg/mL concentration in 1× TBS‐T buffer overnight. After four 7 min wash cycles in prechilled 1× TBS‐T, the blots were incubated for 1.5 h with a horse anti‐mouse (#7076; Cell Signaling, Danvers, MA) or a goat anti‐rabbit HRP‐linked secondary antibody (#31460; Thermo Fisher Scientific) used at a dilution of 1:10000 or 1:40000, respectively. After four 5 min‐long blot wash cycles in 1X TBS‐T and a final wash in dH_2_O, the chemiluminescent signal was developed for 5 min in a 1:1 mixture of SuperSignal West Pico PLUS (Thermo Fisher Scientific) and West Dura (Thermo Fisher Scientific) chemiluminescent substrates (Thermo Fisher Scientific). Protein bands were visualized using ChemiDoc MP imaging system (Bio‐Rad). Band intensity after ~4 min exposure time was analyzed using GelAnalyzer v.23.1 software using the valley‐to‐valley background detection method.

### 2.19 Assessment of Skeletal Muscle Oxidative Stress

Oxidative stress and cellular damage was assessed via conventional Western blotting method by detecting 4‐hydroxynonenal (4‐HNE), an aldehyde produced from the peroxidation of omega‐6 fatty acids. TA muscles were lysed by polytron homogenization using a mixture of 10× RIPA lysis buffer, phosphatase inhibitor (PhosSTOP; Roche, Sigma‐Aldrich Inc.), protease inhibitor (Complete Mini; Rosche. Sigma‐Aldrich Inc.) and PMSF. Under standard SDS‐PAGE conditions, proteins were separated on a 10% polyacrylamide gel. Nitrocellulose membranes were incubated with anti‐4HNE (ab46545, 1:1000; Abcam).

### 2.20 Strength Training

An electrical stimulation‐based model of strength training was developed using the Aurora 1300A system (Aurora, ON, Canada). For both isometric force training and testing, WT mice were anesthetized using 5% isoflurane (3% maintenance). The knee was secured, and needle electrodes were inserted in the gluteal muscle and quadriceps muscle to allow for stimulation of the sciatic nerve. The foot was secured with surgical tape to the foot pedal and a single test stimulation was used to ensure adequate sciatic nerve stimulation. The foot angle was offset to +15° and the muscles allowed to acclimate to this position for at least 30 s. This angle was chosen to adequately stretch the Achilles tendon and plantarflexors but also protect against potential muscle damage due to excessive stretch during the eccentric portion of the repetition. The current used for training and testing was 25 mA. For training, the following protocol was used: 0.1 s delay establishing a baseline tension prior to each contraction, 0.5 s of 80 Hz stimulation during which the ankle angle changes at a constant rate from +15° to −19° (concentric), 0.2 s isometric hold at −19° with 80 Hz stimulation, 0.5 s of 40 Hz stimulation where the ankle rotated from −19° back to +15° (eccentric), and a 0.7 s rest at +15° before the next repetition. Mice completed 4 sets of 10 stimulations, with 2 min rest between sets. Mice were anesthetized for less than 15 min during each session. Mice were trained twice per week for 4 weeks. Maximal isometric force measurements were obtained two days prior to the first training session and immediately prior to the last training session. Mice were euthanized immediately after the last training session following an overnight fasting. Body and muscle weights were recorded, and blood was collected via cardiac puncture. EVs were isolated and characterized as outlined above.

### 2.21 Statistical Data Analysis

Data was analyzed using GraphPad Prism (v.10.1.2). Student's *t*‐test was used to detect differences between two groups, whereas one‐way ANOVA and Fisher's LSD or Tukey's test were used to detect differences between multiple groups. Two‐way ANOVA was used to detect immobilization × GPX1 LP interaction and main effects, followed by Tukey's *post hoc* analysis. Differences were considered significant at *p* < 0.05.

## 3 Results

### 3.1 Validation of Endurance Exercise Training in Mice

Donor mice ran an average distance of 20 km by the end of the 4‐week exercise training period (Figure S1a–c), which resulted in a decrease in body weight and significant increases in relative skeletal muscle weight, Type IIb to IIa fiber shift, and capillarization in gastrocnemius muscle (Figure S1d–k). Immediately after the last bout of exercise training, blood samples were obtained from sedentary and exercised animals.

### 3.2 The Impact of Exercise Training on EV Quantity, Size, and Protein Content

**FIGURE 1:**
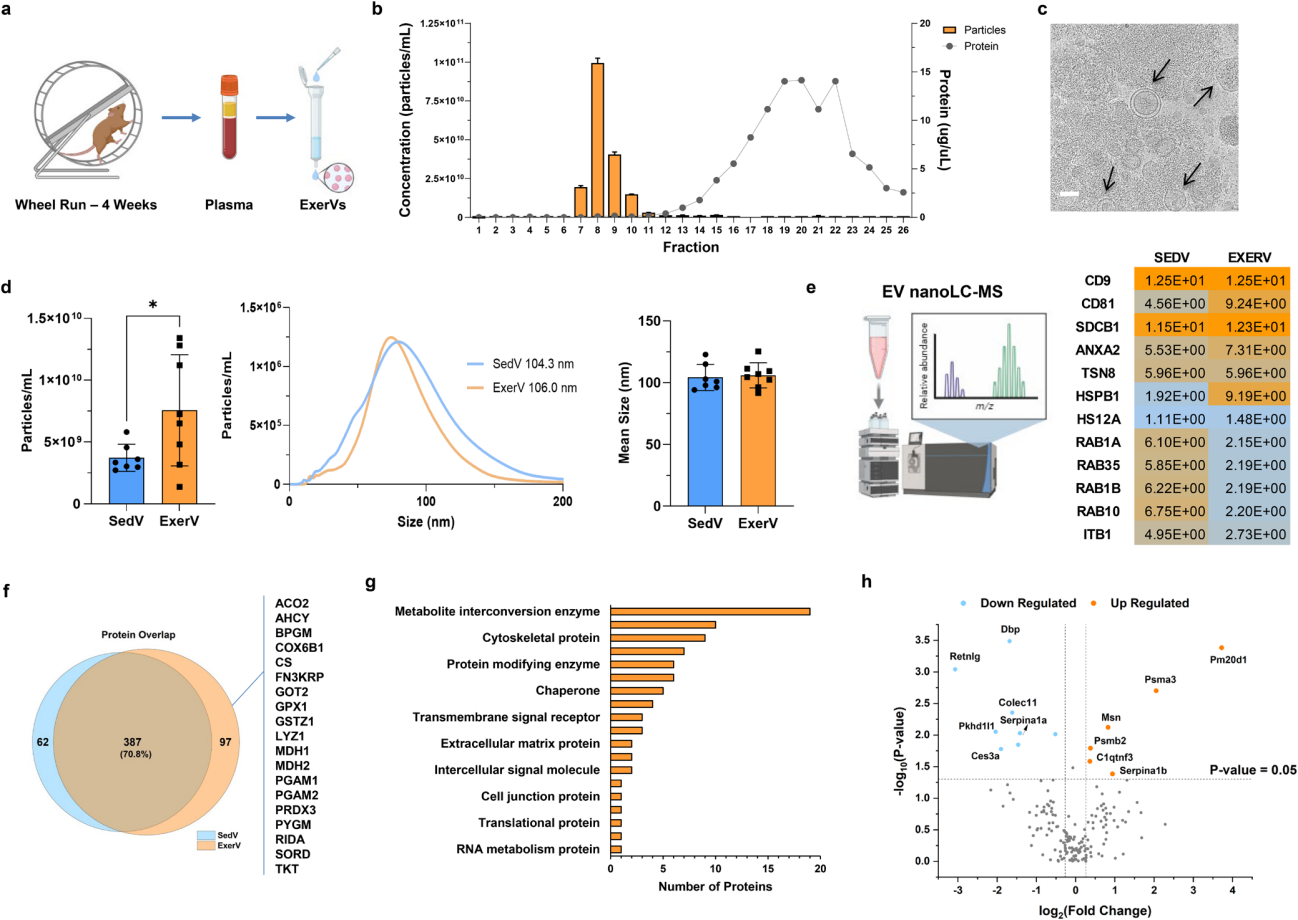
Characterization of EVs Isolated from Blood Plasma After 4 Weeks of Endurance Exercise Training (ExerVs). (a) Schematic diagram of experimental design. ExerVs were isolated from mice (C57BL/6J, 4‐month‐old, male) immediately after the last bout of wheel run training via size exclusion chromatography (SEC) and ultrafiltration (UF). (b) Identification of EVs in Fractions #7–10. (c) Cryo‐EM analysis of ExerVs. (d) NTA analysis of EVs collected after exercise (ExerV) or sedentary conditions (SedV), including concentration, size distribution and mean size. (e) EV markers detected by nanoLC‐MS (orange = increase, blue = decrease). (f) EV proteins identified by nanoLC‐MS, including the list of metabolite interconversion enzymes exclusively present in ExerVs. (g) Identification of ExerV protein families based on PANTHER classification. (h) Volcano plot showing the up‐regulated (orange) and down‐regulated (blue) proteins, *p* < 0.05. PM20D1 and DBP only, Q < 0.05. *n* = 7–8 mice/group for characterization. *n* = 4–5 mice/group for proteomic analysis. All values expressed as mean ± SD. *p < 0.05. Scale bar: 50 nm.

EVs (SedVs and ExerVs) were isolated from plasma via size exclusion chromatography (SEC) (Figure 1a,b) and characterized by nanoparticle tracking analysis (NTA), bicinchoninic acid (BCA) assay, cryogenic electron microscopy (Cryo‐EM), and mass spectroscopy (nanoLC‐MS). EVs were detected in Fractions #7–10 of SEC (Figure 1b), and EVs possessed a bilipid layer and intercellular material based on Cryo‐EM imaging (Figure 1c). A significant increase in ExerV concentration was detected immediately after the last bout of exercise post‐training, yet no difference in the mean size was observed between SedVs and ExerVs (SedV = 104 nm, ExerV = 106 nm) (Figure 1d). 546 proteins were identified in SedVs and ExerVs, including several EV‐associated markers (CD9, CD81, SDCB1, ANXA2, TSN8, HSPB1, HS12A, RABs, ITB1) (Figure 1e). 62 proteins were unique to SedVs, whereas 97 proteins were unique to ExerVs (Figure 1f, Tables S1 and S2). “Metabolite Interconversion Enzyme” was the most represented class for proteins uniquely enriched in ExerVs, specifically “Oxidoreductases” (Figure 1g; list provided in Figure 1f), which included antioxidants glutathione peroxidase 1 (GPX1), peroxiredoxin 3 (PRDX3), and glutathione S‐transferase zeta 1 (GSTZ1). This class was significantly overrepresented (2.08‐fold enrichment; Raw *p* = 0.0023; FDR = 0.042). Of the shared proteins in SedVs and ExerVs, 7 proteins were significantly upregulated (Q < 0.05: PM20D1; *p* < 0.05: PSMA3, EZR, RDX, MSN, C1QTNF3, and SERPINA1B) and 8 were significantly downregulated (Q < 0.05: DBP; p < 0.05: RETNLG, FN1, COLEC11, SERPINA1A, PKHD1L1, A2M, and CES3A) in ExerVs (Figure 1h, Table S3). Classic exerkines [6] were not detected in EVs by nanoLC‐MS analysis after 4 weeks of training. However, antioxidants (PRDX2, SOD1, GPX3), adiponectin (ADIPOQ), heat shock proteins (HSP2A, HSP8, HSP12A), and laminin subunits (LAMA2, LAMC1, LAMB1) were detected in both SedVs and ExerVs (Table S3). Overall, these data suggest that endurance exercise training increases the quantity of EVs in plasma, which are uniquely enriched in oxidoreductases, specifically antioxidants GPX1, PRDX3, and GSTZ1.

### 3.3 Systemic Injection of ExerVs Enhances Capillary Density and Blood Perfusion in Skeletal Muscle of Sedentary Mice

**FIGURE 2:**
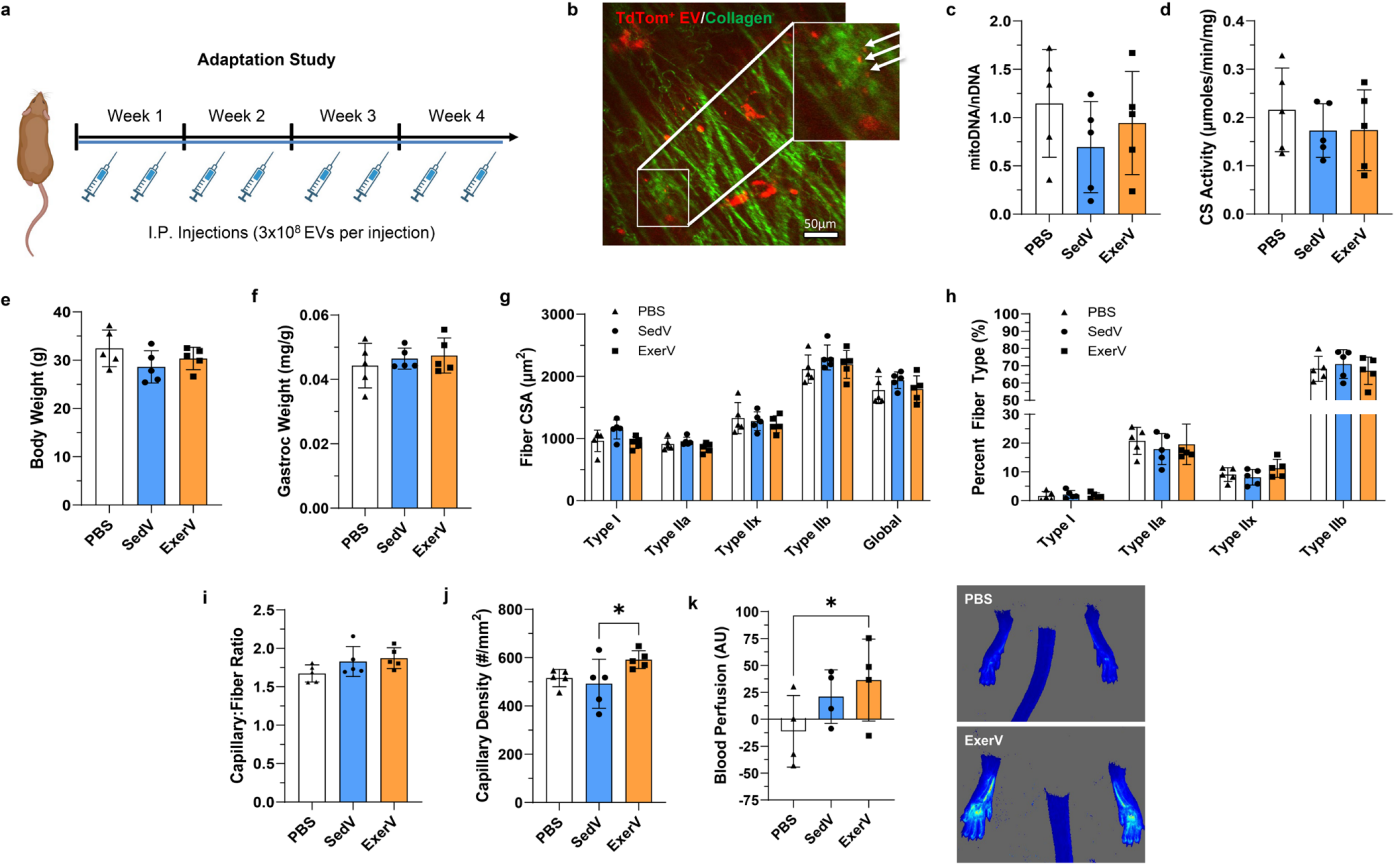
ExerVs Stimulate Capillary Density and Perfusion In Vivo. (a) Schematic diagram of experimental design for ExerV injections. Blood plasma was collected and EVs were isolated from mice after sedentary conditions (SedV) or 4 weeks of wheel run training (ExerV). PBS (control), SedVs or ExerVs were intraperitoneally (i.p.) injected into recipient mice (C57BL/6J, 4‐month‐old, male) twice per week for 4 weeks, then gastrocnemius muscles were evaluated. (b) Validation of tdTomato EVs in gastrocnemius muscle after i.p. injection by broadband multiphoton imaging. Collagen fibers are visualized by their second harmonic generation signal rendered in green. (c) Mitochondrial content. (d) Citrate synthase activity. (e) Body weight. (f) Relative muscle weight. (g) Fiber size (CSA). (h) Fiber type. (i) Capillary content (capillary:Fiber ratio), (j) Capillary density, and (j) Blood perfusion via laser speckle contrast imaging. *n* = 4–5 mice/group. All values expressed as mean ± SD. *p < 0.05.

EVs characterized from donor mice were then intraperitoneally (i.p.) injected into sedentary mice two times per week for 4 weeks to examine the impact on skeletal muscle adaptation (Figure 2a). To validate the delivery of EVs to muscle, plasma‐derived tdTomato^+^ EVs from sedentary mice were i.p. injected and subsequently identified in skeletal muscle of recipient mice using multimodal multiphoton imaging (Figure 2b). Following validation of delivery, skeletal muscle adaptations were assessed after repeated injections of SedVs or ExerVs. No changes in mitochondrial content (mitoDNA/nDNA), mitochondrial function (assessed by citrate synthase (CS) activity), body weight, relative muscle (gastrocnemius) weight, fiber size, fiber type composition, or capillary content (normalized to # of fibers; ANOVA *p* = 0.1) were observed (Figure 2c–i). However, ExerVs significantly increased capillary density in skeletal muscle compared to SedVs (Figure 2j), and ExerVs increased paw blood perfusion compared to PBS treatment (Figure 2k).

**FIGURE 3:**
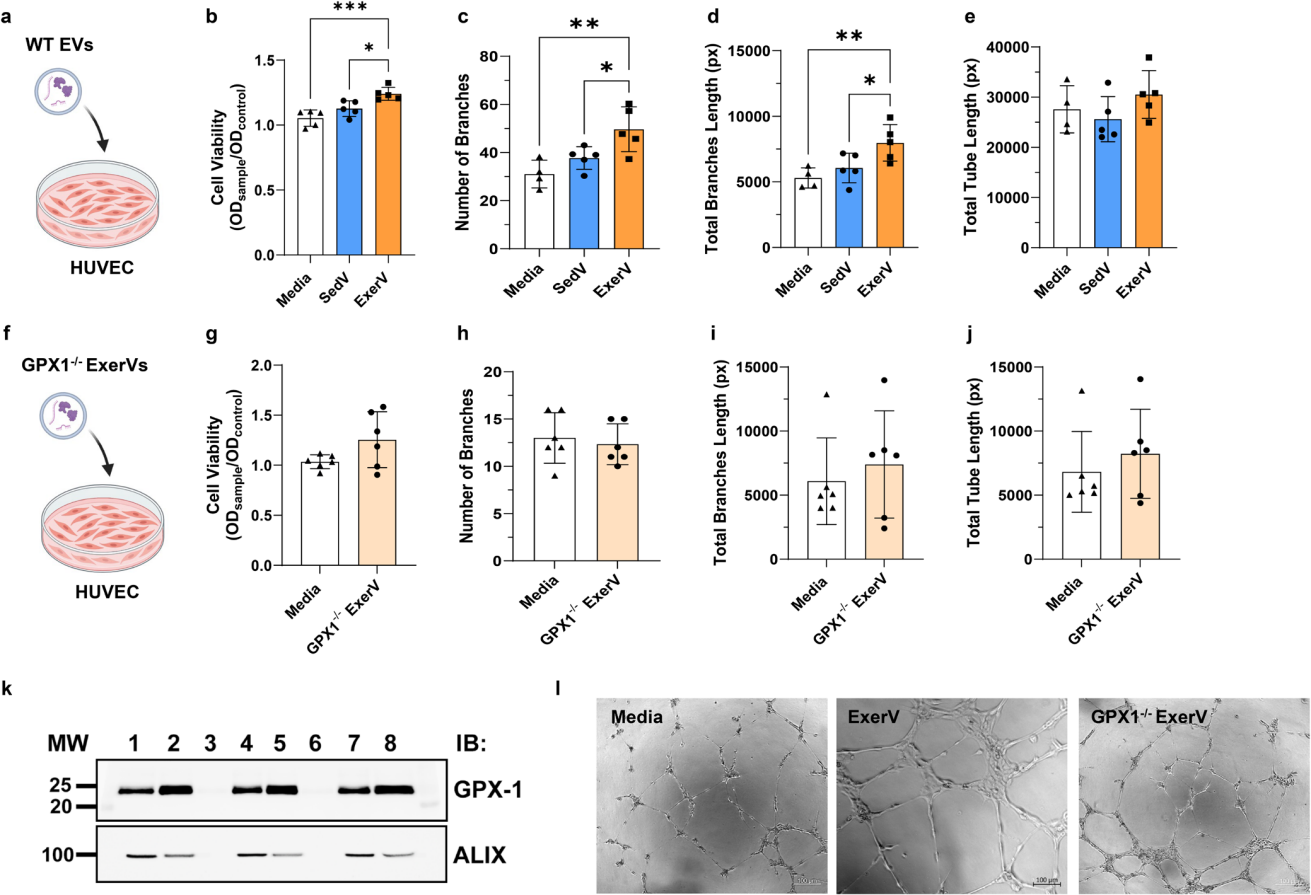
ExerVs Stimulate Vascular Growth In Vitro. (a) Schematic diagram of experimental design. Blood plasma was collected and EVs were isolated from mice after sedentary conditions (SedV) or 4 week of wheel run training (ExerV). Media (control), SedVs and ExerVs were applied to human umbilical vein endothelial cells (HUVEC) in culture on Matrigel and examined for (b) viability, (c) number of branches, (d) total branches length, and (e) total tube length. (f‐j, l) Experiments were repeated using SedVs and ExerVs collected from GPX1^−/−^ mice. (k) Validation of GPX1 content in EVs via multistrip Western blotting. Lanes #1,4,7 = SedVs, Lanes #2,5,8 = ExerVs. *n* = 4–6 mice/group. All values expressed as mean ± SD. *p < 0.05, **p < 0.01, ***p < 0.001. Scale bar: 100 μm.

To test the ability for ExerVs to directly impact endothelial cell structure and function, ExerVs were applied to HUVECs in culture (Figure 3). ExerVs increased cell viability, the number of branches, and branch length compared to both media and SedVs (Figure 3b–e). Given the role for GPX1 in the regulation of vascular growth and integrity [34, 35, 36, 37, 38, 39], ExerVs were isolated from GPX1^−/−^ mice, then applied to HUVECs (Figure 3f). GPX1^−/−^ mice ran an average daily distance of 9.4 km (263 km total distance over 4 week), which is similar to the range for wild type mice (4–12 km/night). Unlike WT ExerVs, GPX1^−/−^ ExerVs had no significant effect on viability, branching, or tube length (Figure 3g–j,l). Western blotting validated enrichment of GPX1 protein in EVs, with (2.98‐fold) or without (1.5‐fold) normalization to ALIX (Figure 3k). Overall, these data suggest that circulating ExerVs may promote vascular growth in a GPX1‐dependent manner.

### 3.4 Direct Injection of GPX1‐Rich ExerVs Accelerates Recovery of Capillary Content in Muscle After Disuse

**FIGURE 4:**
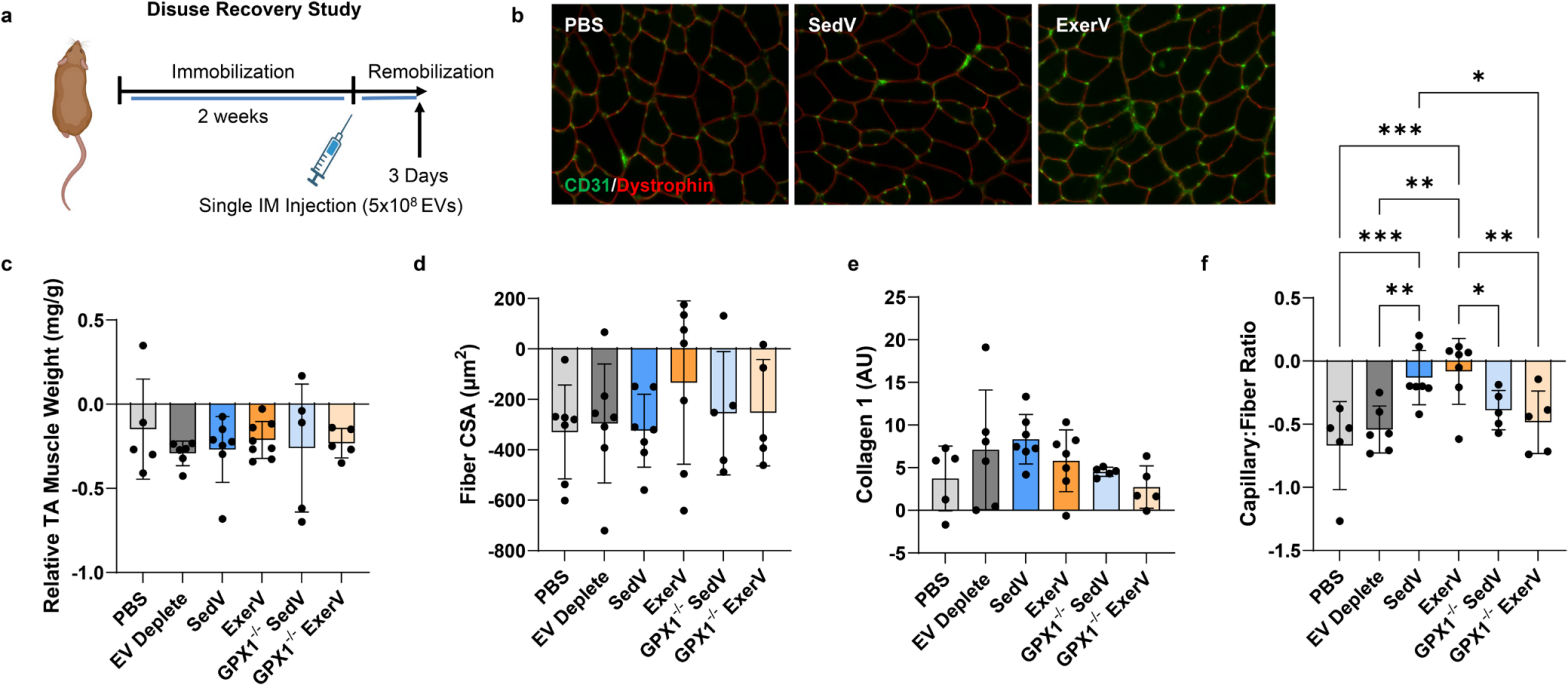
Intramuscular Injection of ExerVs Improves Vascular Growth After Limb Immobilization. (a) Schematic diagram of experimental design. Blood plasma was collected and EVs were isolated from donor WT and GPX1^−/−^ mice after sedentary conditions (SedV) or 4 weeks of wheel run training (ExerV). Protein‐enriched EV‐depleted plasma was also collected from WT mice after training (EV Deplete). Recipient mice were then subjected to 2 weeks of unilateral limb immobilization, followed by a single intramuscular injection of PBS (control), SedVs, ExerVs, or EV depleted plasma prior to remobilization and recovery for 3 days. (b) Representative images of CD31^+^ capillaries in tibialis anterior (TA) skeletal muscle with PBS, WT SedV, and WT ExerV treatments. The following assessments were performed: (c) relative TA muscle weight, (d) fiber size (CSA), (e) collagen 1 accumulation, and (f) capillary content (capillary:Fiber ratio). *n* = 5 mice/group. All values expressed as mean ± SD. *p < 0.05, **p < 0.01, ***p < 0.001.

Recovery of skeletal muscle is frequently delayed after a period of disuse due to loss of mitochondria, mitochondrial dysfunction, and subsequent production of ROS [26, 27, 40, 41, 42, 43]. Therefore, antioxidant‐rich ExerVs were also evaluated for their ability to accelerate recovery of muscle mass, myofiber size, vascular content, and collagen turnover compared to control treatments (PBS, EV‐depleted plasma, SedVs, GPX1^−/−^ SedVs and GPX1^−/−^ ExerVs) (Figure 4a). Whereas a single intramuscular injection of ExerVs did not impact TA weight (Figure 4c), fiber CSA (Figure 4d), or collagen turnover (Figure 4e), capillary content was nearly restored to mobile control leg values (Figure 4f). SedVs also recovered capillary content (Figure 4f). In contrast, EV‐depleted plasma and GPX1^−/−^ EVs did not confer any benefit compared to the PBS control group.

**FIGURE 5:**
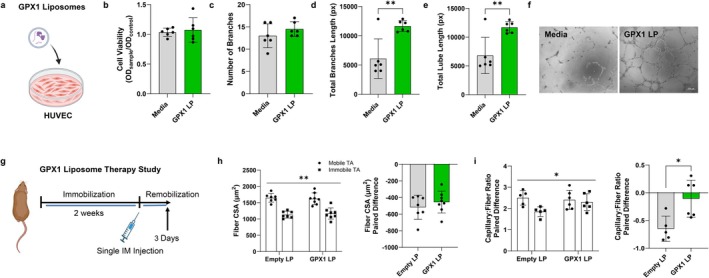
GPX1 Liposomes Stimulate Vascular Growth In Vitro and In Vivo. (a) GPX1 liposomes (LPs) were applied to HUVECs in culture and assessed for (b) viability, (c) number of branches, (d) total branches length, and (e) total tube length. (f) Representative images of HUVEC tube formation with addition of GPX1 LPs. (g) Schematic diagram of experimental design. GPX1 LPs were injected into skeletal muscle after 2 weeks of disuse, immediately prior to remobilization for 3 days, and evaluated for (h) recovery of fiber size (CSA) and (i) capillary content (capillary:Fiber ratio). Each individual data point is shown (left), as well as the differences between the mobile (control) and immobilized limbs within each mouse (right). *n* = 7–8 for LPs. All values expressed as mean ± SD. *p < 0.05, **p < 0.01. Scale bar: 50 μm.

To further establish a role for GPX1 in the promotion of capillary growth after disuse, GPX1 protein was encapsulated in liposomes (GPX1 LP) and either applied to endothelial cells in culture (Figure 5a–f) or injected into skeletal muscle after disuse (Figure 5g–i). GPX1 LPs did not significantly impact viability or branch formation in culture, but potently increased branch length (Figure 5e,f). Similar to WT ExerVs, GPX1 LPs did not recover fiber size, but alleviated capillary refraction compared to control (Empty LPs) (Figure 5h,i). Despite the significant effect of GPX1 LPs on capillary content in vivo, no changes in ROS based on 4‐HNE were detected via Western blotting in whole muscle lysates (LP = 1.00, GPX1 LP = 1.043; *n* = 6/group, *p* = 0.81). Altogether, these data suggest that plasma ExerVs stimulate vascular growth in the context of disuse recovery, and this important outcome may be dependent on GPX1.

### 3.5 Characterization of ExerVs Obtained From Plasma After Resistance Exercise Training

A direct comparison of plasma ExerVs obtained after endurance exercise compared to resistance exercise has not been previously reported in mice. Using an electrical stimulation‐based model for strength training (Figure S2a,b), blood was collected and isolated plasma EVs were evaluated by NTA, BCA, and nanoLC‐MS (Figure S2c–i). In contrast to the increase in plasma ExerV concentration observed after endurance training, a significant decrease in plasma ExerV concentration was detected immediately after the last bout of strength training (Figure S2e). No significant difference in mean size was observed (SedV = 96 nm, ExerV = 109 nm) (Figure S2f). 299 proteins were present in SedVs and ExerVs, including several EV markers (CD9, CD81, SDCB1, ANXA2, HSPB1, RABs, ITB1). 37 proteins were uniquely detected in SedVs, whereas 41 proteins were uniquely detected in ExerVs (Figure S2g). “Protein‐binding activity modulator” was the most represented class for proteins enriched in ExerVs (Figure S2h; list provided in Figure S2g), which included several RAB proteins and HSP90AA1. Of the shared proteins in SedVs and ExerVs, 8 proteins were significantly upregulated (CD5L, F13B, LAMB1, LAMA2, GSN, PLG, NOTCH3, COMP) and 2 were significantly downregulated (RETNLG, MBL1) in ExerVs (*p* < 0.05). Interestingly, “Metabolite interconversion enzyme”, specifically “Oxidoreductase”, was the most represented protein class among proteins identified in both SedVs and ExerVs, which included antioxidants (GPX3, GPX5, SOD1, CAT, GSTP1, PRDX1, PRDX2), flavin reductases (NADPH, BLVRB), and glycogen phosphorylase (PYGB, PYGM, PYGL). Importantly, antioxidants GPX1 and PRDX3, which can be found in mitochondria, were not detected in EVs isolated from plasma after repeated periods of intense contractions. These data suggest plasma ExerVs collected after different exercise modalities may be functionally distinct based on differences in proteomic profiles, yet further analyses are necessary to address this question, which is outside the scope of the current study.

## 4 Discussion

The purpose of this study was to comprehensively examine the quantity, protein content, and function of EVs isolated from blood after multiple bouts of endurance exercise in mice. Here we report that 4 weeks of voluntary wheel running elicits a significant increase in ExerV quantity when blood is collected immediately after the last bout of exercise. These ExerVs are enriched in antioxidants and facilitate capillary growth in vitro and in vivo. Given the enrichment of GPX1 in ExerVs and the well‐established role for GPX1 in vascular integrity [36], experiments were conducted to determine the extent to which ExerV‐mediated effects were dependent on GPX1. GPX1^−/−^ ExerVs demonstrated no benefits, whereas GPX1‐encapsulated liposomes robustly promoted vessel growth in vitro and in vivo. Thus, this study demonstrates that ExerVs are important regulators of vascular remodeling in skeletal muscle and may represent a primary mechanism by which exercise training protects the body against vascular disease.

Several studies have reported an increase in EV quantity in blood after an acute bout of high intensity exercise in humans and mice, without any changes in EV size ([13, 14, 15, 16, 17, 18, 19, 20, 21]). Here we demonstrate that EVs also transiently accumulate in blood post‐exercise after a period of training. Lisi et al. [44] reported no increase in EV quantity after exercise in trained men, yet these samples were collected outside the optimal 1–2‐h post‐exercise window. The primary cell origin and mechanism by which exercise facilitates EV release into blood is not clear, but in vitro analyses have demonstrated that ROS can induce lysosomal dysfunction, ultimately redirecting multivesicular bodies (MVBs) toward the membrane for fusion and release of intraluminal vesicles as EVs [45, 46]. The fact that an acute bout of exercise can increase mitochondrial and membrane‐localized ROS in a variety of cell types [47, 48] suggests that each bout may result in a temporary phase of lysosomal stress and subsequent bolus release of EVs, which theoretically would not be further enhanced with non‐progressive training. Our results are congruent with this hypothesis, as training resulted in a two‐fold increase in plasma ExerV quantity, similar to acute studies.

Transient ROS can also initiate intracellular signaling pathways, including AMP‐activated protein kinase (AMPK) and downstream co‐transcriptional activation of peroxisome proliferator‐activated receptor gamma coactivator‐1α (PGC‐1α) [49], which can increase mitochondrial biogenesis and antioxidant synthesis [50]. The enhancement of mitochondrial‐localized protective factors may be captured by MVBs within the ESCRT pathway, leading to the enrichment of antioxidants consistently observed in plasma ExerVs. This idea is supported by in vitro analyses demonstrating that PGC‐1α overexpression in myotubes increases the secretion of EVs possessing antioxidant mRNAs, including non‐specific glutathione peroxidases [51]. It is tempting to speculate that the repeated induction of PGC‐1α with training results in ExerVs with superior capacity to phenocopy the effects of exercise training. Further studies directly comparing ExerV molecular cargo and function after single versus multiple bouts of exercise are necessary to address this important question.

Acute exercise studies have reported enhanced antioxidant protein in EVs post‐exercise, specifically detecting glutathione peroxidase 4 (GPX4) [20], peroxiredoxin 2 (PRDX2) [17], peroxiredoxin 6 (PRDX6) [20], catalase (CAT) [17, 20, 22], and SOD3 [23]. Interestingly, GPX1 and PRDX3, antioxidants associated with mitochondria [52, 53], were uniquely expressed in our ExerV proteomic dataset after endurance exercise training. In addition, PM20D1, a secreted factor that serves as a UCP‐independent uncoupler of mitochondrial respiration in brown fat [54, 55], was the only protein significantly upregulated in ExerVs compared to SedVs (Q‐value < 0.05). These findings strengthen the hypothesis that repeated bouts of exercise result in adaptive changes that are reflected in EV cargo and suggest that GPX1, PRDX3, and/or PM20D1 may be uniquely endowed with capacity to systemically control redox balance and provide resilience to stress in distal tissues.

Few studies have examined the ability for ExerVs collected after acute or repeated bouts of exercise to initiate beneficial adaptations in mice. Thus, ExerVs were administered to sedentary mice in the current study to examine their impact on skeletal muscle structure and function. No changes in body weight, muscle fiber composition or size were noted, as well as mitochondrial content or function. Unexpectedly, an increase in capillary density was observed, which was associated with increased blood perfusion. Whether the relatively small increase in capillary density is responsible for enhanced perfusion cannot be discerned, but a recent study reported that muscle‐derived ExerVs can directly reduce arterial stiffness in a mouse model of atherosclerosis [56]. Regardless, ExerVs added to HUVEC cells in culture directly induced tube growth, and these results are consistent with similar studies [23, 51]. The mechanistic basis by which ExerVs can promote vessel growth under homeostatic conditions is not clear, but EVs can transfer ROS‐producing enzymes (NOX) and oxidized substrates that can stimulate endothelial cell turnover and outgrowth [57, 58]. The critical balance of pro‐ and antioxidant cargo in SedVs versus ExerVs may ultimately dictate vascular remodeling and fate, particularly under varying conditions (homeostasis vs. disease, aging, disuse recovery). It would be of interest to examine ROS‐producing enzymes and other sources of ROS in EVs post‐exercise, as these factors may provide a significant contribution to growth‐promoting signaling pathways in balance with antioxidant cargo, in a variety of tissues.

GPX1 is expressed in numerous cells and tissues, yet mouse models of GPX1 deficiency demonstrate an essential role for GPX1 in the regulation of vascular growth and integrity [34, 35, 36, 37, 38, 39]. Here we demonstrate using plasma EVs isolated from GPX1^−/−^ mice that GPX1 may be a critical regulator of ExerV‐mediated angiogenesis. Based on these results, we designed and injected liposomes encapsulated with GPX1, which robustly rescued capillary content in skeletal muscle and increased endothelial cell tube formation in culture. These results are similar to Abdelsaid et al. [23] which demonstrated that SOD3 overexpressing EVs induced endothelial cell migration and tube formation in vitro via a vascular endothelial growth factor receptor 2 (VEGFR2)‐mediated signaling event. We did not detect SOD3 in our proteomic analyses and it is not clear how GPX1 may directly stimulate angiogenesis. However, GPX1 has been shown to activate the PI3K/AKT pathway in cancer cells [59], highlighting an alternative antioxidant‐independent mechanism for GPX1 in the induction of growth. Further studies are required to fully understand how GPX1 impacts endothelial cell function.

There are several limitations of this study that should be noted. First, we have not established the cellular origin for ExerVs as a result of endurance exercise training. The unique enrichment of GPX1 with training compared to acute exercise, as well as the enhancement of GPX1 in vascular cells with exercise and PGC‐1α induction [38, 60], suggests that ExerVs may predominantly originate from endothelial cells or vascular mural cells (pericytes, smooth muscle cells). This would be logical given the ability for shear stress to activate ROS in endothelial cells during exercise [61] and readily release EVs into the bloodstream. Cell‐specific lineage tracing and plasma EV flow cytometry experiments will be necessary to address this question. Second, SedVs and ExerVs recovered capillary content to the same extent in skeletal muscle in response to disuse. Both SedVs and ExerVs possess antioxidant cargo, including GPX1, and may be equally therapeutic. However, it is possible that ExerVs more effectively neutralized ROS and prevented capillary degradation and during the initial remobilization phase (1 day post‐remobilization), whereas SedVs allowed for recovery from the presence of ROS at 3 days post‐remobilization, resulting in no difference noted between groups. Timing is an important consideration, as any delay in preventing or reducing ROS could have negative impacts on muscle recovery. Evidence for this hypothesis can be found in the beneficial trends in fiber size noted with ExerV treatment compared to SedV, as well as preferential recovery of Type I fibers in our most recent paper [62]. Finally, we did not directly compare the ability for ExerVs collected after training to stimulate angiogenesis to a greater extent than ExerVs collected after an acute bout of endurance exercise. The unique enrichment of GPX1 and other antioxidants (PRDX3, GSTZ1) in this study compared to acute studies suggests differences in functionality, yet further studies are necessary to address this important question.

In conclusion, the results from this study suggest that endurance exercise training can induce the release of GPX1‐enriched ExerVs that are important regulators of skeletal muscle angiogenesis. Thus, ExerVs and/or GPX1‐loaded liposomes may represent novel therapeutic approaches to conditions that require revascularization, such as disuse recovery, ischemia, or aging.

## Author Contributions

Alexander M. Fliflet: conception and design, collection of data, data analysis, writing, approval. Ray A. Spradlin: conception and design, collection of data, data analysis, writing, approval. Yanqi Tan: conception and design, collection of data, data analysis, writing, approval. Ane Nishitha Vijayan: conception and design, collection of data, data analysis, approval. Sung Jun Choi: collection of data, data analysis, approval. Wei‐Chun Kao: collection of data, data analysis, approval. Edita Aksamitiene: conception and design, collection of data, data analysis, writing, approval. Michael Nelappana: collection of data, data analysis, writing, approval. Shengzhe Ding: collection of data, data analysis, writing, approval. Kai‐Yu Huang: collection of data, data analysis, approval. Aryaman Joshi: collection of data, data analysis, approval. Nurila Kambar: collection of data, data analysis, writing, approval. Tom Bludgen: collection of data, approval. Madeleine Meehan: collection of data, approval. Jayna L. Boss: collection of data, approval. Michael Knipp: collection of data, approval. Hongming Fan: conception and design, collection of data, data analysis, writing, approval. Cecilia Leal: data analysis, financial support, approval. Roger A. Sunde: conception and design, approval. Lawrence W. Dobrucki: data analysis, financial support, writing, approval. Stephen Boppart: conception and design, approval. Hyunjoon Kong: conception and design, financial support, writing, approval. Jonathan V. Sweedler: conception and design, data analysis, financial support, writing, approval. Marni D. Boppart: conception and design, data analysis, financial support, writing, approval.

## Funding

This study was supported by NIAMS of the National Institutes of Health under award number R01AR072735 (to MDB), Translational Research Institute for Space Health (TRISH) Award (T0701) under a Cooperative Agreement with NASA (NNX16AO69A) (to MDB), NIH P30 DA018310 (to JVS), and the NIH/NIBIB Center for Label‐free Imaging and Multiscale Biophotonics (CLIMB) (P41EB031772) (to SAB). AMF was supported by a Beckman Institute Graduate Student Fellowship. Cryo‐EM imaging was performed using an instrument partially funded by the National Institutes of Health (NIH) under project P30DA018310, S10OD028700, and vesicle characterization by Cryo‐EM was supported by NIH grant R01GM143723 (to CL).

## Conflicts of Interest

The authors declare no conflicts of interest.

## Supporting information


**Data S1:** Supplementary Figure.

**Data S2:** Supplementary Figure.

**Table S1:** Proteins Unique to ExerVs.

**Table S2:** Proteins Unique to SedVs.

**Table S3:** Proteins Shared Between SedVs and ExerVs.

## Data Availability

Proteomic data were deposited in ProteomeXchange (PXD064533; Token Gxe2kPXu1Eo5).
