# Supplementary figures and images for: Exercise Training Stimulates the Release of Glutathione Peroxidase 1 (GPX1)‐Enriched Extracellular Vesicles That Promote Angiogenesis
Source: FASEB J. 2026 Jun 18;40(12):e72052. doi: 10.1096/fj.202505096RR (PMC13278521; doi:10.1096/fj.202505096RR)

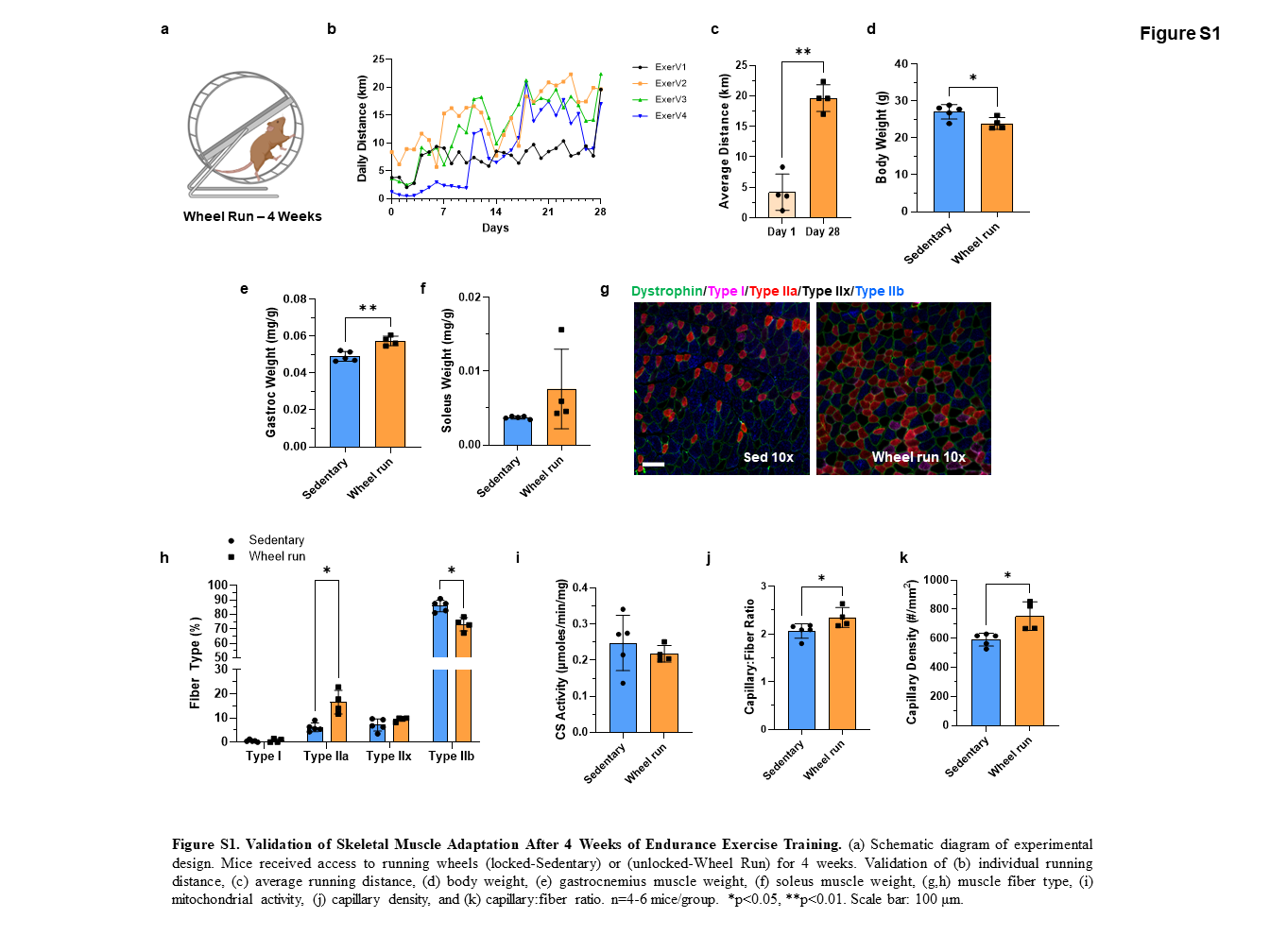

Supplement: Supplementary file 1 — Data S1: Supplementary Figure. [file FSB2-40-e72052-s002.tif]

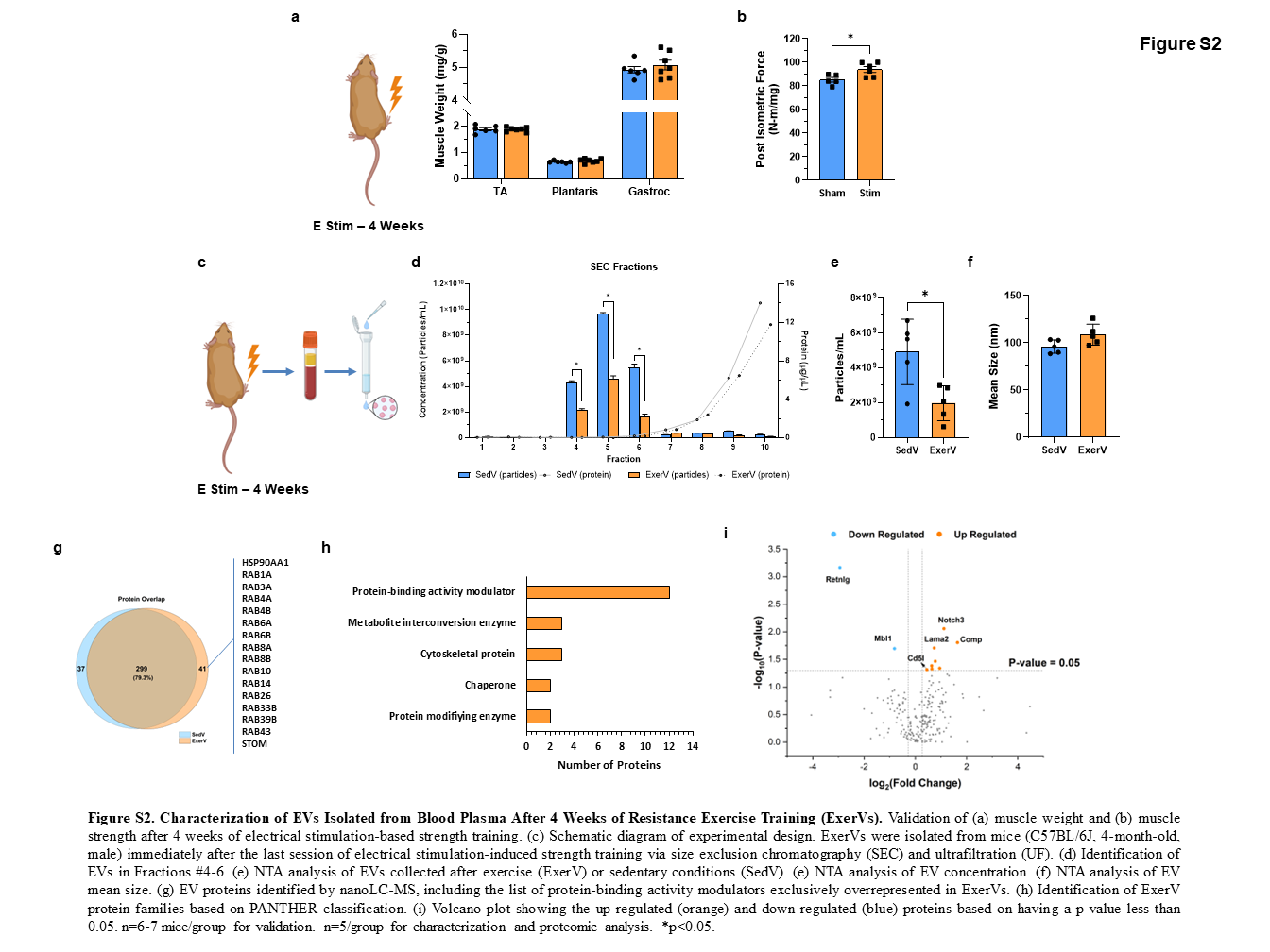

Supplement: Supplementary file 2 — Data S2: Supplementary Figure. [file FSB2-40-e72052-s003.tif]
