# Supplementary material for: Exercise Training Stimulates the Release of Glutathione Peroxidase 1 (GPX1)‐Enriched Extracellular Vesicles That Promote Angiogenesis
Source: FASEB J. 2026 Jun 18;40(12):e72052. doi: 10.1096/fj.202505096RR (PMC13278521; doi:10.1096/fj.202505096RR)
Supplement: Supplementary file 3 — Table S1: Proteins Unique to ExerVs. [file FSB2-40-e72052-s001.docx]

**Supplemental Table 1. Proteins Unique to ExerVs**

| Accession | Description |
| --- | --- |
| Q91ZU6\|DYST_MOUSE | Dystonin OS=Mus musculus OX=10090 GN=Dst PE=1 SV=2 |
| P19096\|FAS_MOUSE | Fatty acid synthase OS=Mus musculus OX=10090 GN=Fasn PE=1 SV=2 |
| Q7TMD7\|DSG4_MOUSE | Desmoglein-4 OS=Mus musculus OX=10090 GN=Dsg4 PE=1 SV=1 |
| Q01149\|CO1A2_MOUSE | Collagen alpha-2(I) chain OS=Mus musculus OX=10090 GN=Col1a2 PE=1 SV=2 |
| Q9WUB3\|PYGM_MOUSE | Glycogen phosphorylase, muscle form OS=Mus musculus OX=10090 GN=Pygm PE=1 SV=3 |
| P29341\|PABP1_MOUSE | Polyadenylate-binding protein 1 OS=Mus musculus OX=10090 GN=Pabpc1 PE=1 SV=2 |
| Q9JHI9\|S40A1_MOUSE | Solute carrier family 40 member 1 OS=Mus musculus OX=10090 GN=Slc40a1 PE=1 SV=1 |
| Q8BSM7\|LAT3_MOUSE | Large neutral amino acids transporter small subunit 3 OS=Mus musculus OX=10090 GN=Slc43a1 PE=1 SV=1 |
| P06683\|CO9_MOUSE | Complement component C9 OS=Mus musculus OX=10090 GN=C9 PE=1 SV=2 |
| Q9Z0M6\|AGRE5_MOUSE | Adhesion G protein-coupled receptor E5 OS=Mus musculus OX=10090 GN=Adgre5 PE=1 SV=2 |
| Q02053\|UBA1_MOUSE | Ubiquitin-like modifier-activating enzyme 1 OS=Mus musculus OX=10090 GN=Uba1 PE=1 SV=1 |
| Q8CC27\|CACB2_MOUSE | Voltage-dependent L-type calcium channel subunit beta-2 OS=Mus musculus OX=10090 GN=Cacnb2 PE=1 SV=1 |
| Q9CZU6\|CISY_MOUSE | Citrate synthase, mitochondrial OS=Mus musculus OX=10090 GN=Cs PE=1 SV=1 |
| P46471\|PRS7_MOUSE | 26S proteasome regulatory subunit 7 OS=Mus musculus OX=10090 GN=Psmc2 PE=1 SV=5 |
| P40142\|TKT_MOUSE | Transketolase OS=Mus musculus OX=10090 GN=Tkt PE=1 SV=1 |
| Q7TMS5\|ABCG2_MOUSE | Broad substrate specificity ATP-binding cassette transporter ABCG2 OS=Mus musculus OX=10090 GN=Abcg2 PE=1 SV=1 |
| P05202\|AATM_MOUSE | Aspartate aminotransferase, mitochondrial OS=Mus musculus OX=10090 GN=Got2 PE=1 SV=1 |
| P14685\|PSMD3_MOUSE | 26S proteasome non-ATPase regulatory subunit 3 OS=Mus musculus OX=10090 GN=Psmd3 PE=1 SV=3 |
| Q9D154\|ILEUA_MOUSE | Leukocyte elastase inhibitor A OS=Mus musculus OX=10090 GN=Serpinb1a PE=1 SV=1 |
| P62874\|GBB1_MOUSE | Guanine nucleotide-binding protein G(I)/G(S)/G(T) subunit beta-1 OS=Mus musculus OX=10090 GN=Gnb1 PE=1 SV=3 |
| P62880\|GBB2_MOUSE | Guanine nucleotide-binding protein G(I)/G(S)/G(T) subunit beta-2 OS=Mus musculus OX=10090 GN=Gnb2 PE=1 SV=3 |
| P50247\|SAHH_MOUSE | Adenosylhomocysteinase OS=Mus musculus OX=10090 GN=Ahcy PE=1 SV=3 |
| P07901\|HS90A_MOUSE | Heat shock protein HSP 90-alpha OS=Mus musculus OX=10090 GN=Hsp90aa1 PE=1 SV=4 |
| P11499\|HS90B_MOUSE | Heat shock protein HSP 90-beta OS=Mus musculus OX=10090 GN=Hsp90ab1 PE=1 SV=3 |
| Q05909\|PTPRG_MOUSE | Receptor-type tyrosine-protein phosphatase gamma OS=Mus musculus OX=10090 GN=Ptprg PE=1 SV=1 |
| P15379\|CD44_MOUSE | CD44 antigen OS=Mus musculus OX=10090 GN=Cd44 PE=1 SV=3 |
| P70441\|NHRF1_MOUSE | Na(+)/H(+) exchange regulatory cofactor NHE-RF1 OS=Mus musculus OX=10090 GN=Slc9a3r1 PE=1 SV=3 |
| O55222\|ILK_MOUSE | Integrin-linked protein kinase OS=Mus musculus OX=10090 GN=Ilk PE=1 SV=2 |
| O88200\|CLC11_MOUSE | C-type lectin domain family 11 member A OS=Mus musculus OX=10090 GN=Clec11a PE=1 SV=1 |
| Q9DBJ1\|PGAM1_MOUSE | Phosphoglycerate mutase 1 OS=Mus musculus OX=10090 GN=Pgam1 PE=1 SV=3 |
| O70250\|PGAM2_MOUSE | Phosphoglycerate mutase 2 OS=Mus musculus OX=10090 GN=Pgam2 PE=1 SV=3 |
| Q99JI4\|PSMD6_MOUSE | 26S proteasome non-ATPase regulatory subunit 6 OS=Mus musculus OX=10090 GN=Psmd6 PE=1 SV=1 |
| Q61292\|LAMB2_MOUSE | Laminin subunit beta-2 OS=Mus musculus OX=10090 GN=Lamb2 PE=1 SV=2 |
| Q8K274\|KT3K_MOUSE | Ketosamine-3-kinase OS=Mus musculus OX=10090 GN=Fn3krp PE=1 SV=2 |
| Q71KU9\|FGL1_MOUSE | Fibrinogen-like protein 1 OS=Mus musculus OX=10090 GN=Fgl1 PE=1 SV=2 |
| Q9WV32\|ARC1B_MOUSE | Actin-related protein 2/3 complex subunit 1B OS=Mus musculus OX=10090 GN=Arpc1b PE=1 SV=4 |
| Q64442\|DHSO_MOUSE | Sorbitol dehydrogenase OS=Mus musculus OX=10090 GN=Sord PE=1 SV=3 |
| Q9JIM1\|S29A1_MOUSE | Equilibrative nucleoside transporter 1 OS=Mus musculus OX=10090 GN=Slc29a1 PE=1 SV=3 |
| Q9QYB1\|CLIC4_MOUSE | Chloride intracellular channel protein 4 OS=Mus musculus OX=10090 GN=Clic4 PE=1 SV=3 |
| Q9JL99\|CLC1B_MOUSE | C-type lectin domain family 1 member B OS=Mus musculus OX=10090 GN=Clec1b PE=1 SV=1 |
| P70274\|SEPP1_MOUSE | Selenoprotein P OS=Mus musculus OX=10090 GN=Selenop PE=1 SV=3 |
| Q6ZQL4\|WDR43_MOUSE | WD repeat-containing protein 43 OS=Mus musculus OX=10090 GN=Wdr43 PE=1 SV=2 |
| P51885\|LUM_MOUSE | Lumican OS=Mus musculus OX=10090 GN=Lum PE=1 SV=2 |
| P08113\|ENPL_MOUSE | Endoplasmin OS=Mus musculus OX=10090 GN=Hsp90b1 PE=1 SV=2 |
| Q8BG32\|PSD11_MOUSE | 26S proteasome non-ATPase regulatory subunit 11 OS=Mus musculus OX=10090 GN=Psmd11 PE=1 SV=3 |
| P61982\|1433G_MOUSE | 14-3-3 protein gamma OS=Mus musculus OX=10090 GN=Ywhag PE=1 SV=2 |
| Q9CQV8\|1433B_MOUSE | 14-3-3 protein beta/alpha OS=Mus musculus OX=10090 GN=Ywhab PE=1 SV=3 |
| Q8VDN2\|AT1A1_MOUSE | Sodium/potassium-transporting ATPase subunit alpha-1 OS=Mus musculus OX=10090 GN=Atp1a1 PE=1 SV=1 |
| Q60932\|VDAC1_MOUSE | Voltage-dependent anion-selective channel protein 1 OS=Mus musculus OX=10090 GN=Vdac1 PE=1 SV=3 |
| Q9ES46\|PARVB_MOUSE | Beta-parvin OS=Mus musculus OX=10090 GN=Parvb PE=1 SV=1 |
| Q9ES89\|EXTL2_MOUSE | Exostosin-like 2 OS=Mus musculus OX=10090 GN=Extl2 PE=1 SV=1 |
| Q64525\|H2B2B_MOUSE | Histone H2B type 2-B OS=Mus musculus OX=10090 GN=Hist2h2bb PE=1 SV=3 |
| Q6ZWY9\|H2B1C_MOUSE | Histone H2B type 1-C/E/G OS=Mus musculus OX=10090 GN=H2bc4 PE=1 SV=3 |
| Q64478\|H2B1H_MOUSE | Histone H2B type 1-H OS=Mus musculus OX=10090 GN=H2bc9 PE=1 SV=3 |
| P10853\|H2B1F_MOUSE | Histone H2B type 1-F/J/L OS=Mus musculus OX=10090 GN=H2bc7 PE=1 SV=2 |
| Q8CGP2\|H2B1P_MOUSE | Histone H2B type 1-P OS=Mus musculus OX=10090 GN=Hist1h2bp PE=1 SV=3 |
| Q64475\|H2B1B_MOUSE | Histone H2B type 1-B OS=Mus musculus OX=10090 GN=H2bc3 PE=1 SV=3 |
| Q8CGP1\|H2B1K_MOUSE | Histone H2B type 1-K OS=Mus musculus OX=10090 GN=H2bc12 PE=1 SV=3 |
| P10854\|H2B1M_MOUSE | Histone H2B type 1-M OS=Mus musculus OX=10090 GN=H2bc14 PE=1 SV=2 |
| O35226\|PSMD4_MOUSE | 26S proteasome non-ATPase regulatory subunit 4 OS=Mus musculus OX=10090 GN=Psmd4 PE=1 SV=1 |
| Q99JW4\|LIMS1_MOUSE | LIM and senescent cell antigen-like-containing domain protein 1 OS=Mus musculus OX=10090 GN=Lims1 PE=1 SV=3 |
| P17897\|LYZ1_MOUSE | Lysozyme C-1 OS=Mus musculus OX=10090 GN=Lyz1 PE=1 SV=1 |
| Q99KI0\|ACON_MOUSE | Aconitate hydratase, mitochondrial OS=Mus musculus OX=10090 GN=Aco2 PE=1 SV=1 |
| P11352\|GPX1_MOUSE | Glutathione peroxidase 1 OS=Mus musculus OX=10090 GN=Gpx1 PE=1 SV=2 |
| P20108\|PRDX3_MOUSE | Thioredoxin-dependent peroxide reductase, mitochondrial OS=Mus musculus OX=10090 GN=Prdx3 PE=1 SV=1 |
| Q61475\|DAF1_MOUSE | Complement decay-accelerating factor, GPI-anchored OS=Mus musculus OX=10090 GN=Cd55 PE=1 SV=2 |
| P57780\|ACTN4_MOUSE | Alpha-actinin-4 OS=Mus musculus OX=10090 GN=Actn4 PE=1 SV=1 |
| Q7TPR4\|ACTN1_MOUSE | Alpha-actinin-1 OS=Mus musculus OX=10090 GN=Actn1 PE=1 SV=1 |
| P51881\|ADT2_MOUSE | ADP/ATP translocase 2 OS=Mus musculus OX=10090 GN=Slc25a5 PE=1 SV=3 |
| P48962\|ADT1_MOUSE | ADP/ATP translocase 1 OS=Mus musculus OX=10090 GN=Slc25a4 PE=1 SV=4 |
| Q9WVL0\|MAAI_MOUSE | Maleylacetoacetate isomerase OS=Mus musculus OX=10090 GN=Gstz1 PE=1 SV=1 |
| O70456\|1433S_MOUSE | 14-3-3 protein sigma OS=Mus musculus OX=10090 GN=Sfn PE=1 SV=2 |
| P0DJI6\|FCOR_MOUSE | Foxo1-corepressor OS=Mus musculus OX=10090 GN=Fcor PE=1 SV=1 |
| Q8K558\|TRML1_MOUSE | Trem-like transcript 1 protein OS=Mus musculus OX=10090 GN=Treml1 PE=1 SV=2 |
| P14152\|MDHC_MOUSE | Malate dehydrogenase, cytoplasmic OS=Mus musculus OX=10090 GN=Mdh1 PE=1 SV=3 |
| Q64433\|CH10_MOUSE | 10 kDa heat shock protein, mitochondrial OS=Mus musculus OX=10090 GN=Hspe1 PE=1 SV=2 |
| O89051\|ITM2B_MOUSE | Integral membrane protein 2B OS=Mus musculus OX=10090 GN=Itm2b PE=1 SV=1 |
| P43277\|H13_MOUSE | Histone H1.3 OS=Mus musculus OX=10090 GN=H1-3 PE=1 SV=2 |
| P56391\|CX6B1_MOUSE | Cytochrome c oxidase subunit 6B1 OS=Mus musculus OX=10090 GN=Cox6b1 PE=1 SV=2 |
| P15864\|H12_MOUSE | Histone H1.2 OS=Mus musculus OX=10090 GN=H1-2 PE=1 SV=2 |
| P08249\|MDHM_MOUSE | Malate dehydrogenase, mitochondrial OS=Mus musculus OX=10090 GN=Mdh2 PE=1 SV=3 |
| P99027\|RLA2_MOUSE | 60S acidic ribosomal protein P2 OS=Mus musculus OX=10090 GN=Rplp2 PE=1 SV=3 |
| O88342\|WDR1_MOUSE | WD repeat-containing protein 1 OS=Mus musculus OX=10090 GN=Wdr1 PE=1 SV=3 |
| P52760\|RIDA_MOUSE | 2-iminobutanoate/2-iminopropanoate deaminase OS=Mus musculus OX=10090 GN=Rida PE=1 SV=3 |
| P62259\|1433E_MOUSE | 14-3-3 protein epsilon OS=Mus musculus OX=10090 GN=Ywhae PE=1 SV=1 |
| Q9JHK5\|PLEK_MOUSE | Pleckstrin OS=Mus musculus OX=10090 GN=Plek PE=1 SV=1 |
| Q99LX0\|PARK7_MOUSE | Parkinson disease protein 7 homolog OS=Mus musculus OX=10090 GN=Park7 PE=1 SV=1 |
| P10404\|ENV1_MOUSE | MLV-related proviral Env polyprotein OS=Mus musculus OX=10090 PE=1 SV=3 |
| D7PDD4\|G6B_MOUSE | Megakaryocyte and platelet inhibitory receptor G6b OS=Mus musculus OX=10090 GN=Mpig6b PE=1 SV=1 |
| Q61599\|GDIR2_MOUSE | Rho GDP-dissociation inhibitor 2 OS=Mus musculus OX=10090 GN=Arhgdib PE=1 SV=3 |
| Q01730\|RSU1_MOUSE | Ras suppressor protein 1 OS=Mus musculus OX=10090 GN=Rsu1 PE=1 SV=3 |
| P29391\|FRIL1_MOUSE | Ferritin light chain 1 OS=Mus musculus OX=10090 GN=Ftl1 PE=1 SV=2 |
| P15327\|PMGE_MOUSE | Bisphosphoglycerate mutase OS=Mus musculus OX=10090 GN=Bpgm PE=1 SV=2 |
| Q78HU7\|GLPC_MOUSE | Glycophorin-C OS=Mus musculus OX=10090 GN=Gypc PE=1 SV=2 |
| P09103\|PDIA1_MOUSE | Protein disulfide-isomerase OS=Mus musculus OX=10090 GN=P4hb PE=1 SV=2 |
| P18760\|COF1_MOUSE | Cofilin-1 OS=Mus musculus OX=10090 GN=Cfl1 PE=1 SV=3 |
| P20065\|TYB4_MOUSE | Thymosin beta-4 OS=Mus musculus OX=10090 GN=Tmsb4x PE=1 SV=1 |
