# Supplementary material for: Exercise Training Stimulates the Release of Glutathione Peroxidase 1 (GPX1)‐Enriched Extracellular Vesicles That Promote Angiogenesis
Source: FASEB J. 2026 Jun 18;40(12):e72052. doi: 10.1096/fj.202505096RR (PMC13278521; doi:10.1096/fj.202505096RR)
Supplement: Supplementary file 4 — Table S2: Proteins Unique to SedVs. [file FSB2-40-e72052-s004.docx]

**Supplemental Table 2. Proteins Unique to SedVs**

| Accession | Description |
| --- | --- |
| Q8R0W0\|EPIPL_MOUSE | Epiplakin OS=Mus musculus OX=10090 GN=Eppk1 PE=1 SV=2 |
| P11881\|ITPR1_MOUSE | Inositol 1,4,5-trisphosphate receptor type 1 OS=Mus musculus OX=10090 GN=Itpr1 PE=1 SV=2 |
| Q9Z329\|ITPR2_MOUSE | Inositol 1,4,5-trisphosphate receptor type 2 OS=Mus musculus OX=10090 GN=Itpr2 PE=1 SV=4 |
| P70227\|ITPR3_MOUSE | Inositol 1,4,5-trisphosphate receptor type 3 OS=Mus musculus OX=10090 GN=Itpr3 PE=1 SV=3 |
| Q61554\|FBN1_MOUSE | Fibrillin-1 OS=Mus musculus OX=10090 GN=Fbn1 PE=1 SV=2 |
| P97412\|LYST_MOUSE | Lysosomal-trafficking regulator OS=Mus musculus OX=10090 GN=Lyst PE=1 SV=1 |
| P97789\|XRN1_MOUSE | 5'-3' exoribonuclease 1 OS=Mus musculus OX=10090 GN=Xrn1 PE=1 SV=1 |
| Q9WTS4\|TEN1_MOUSE | Teneurin-1 OS=Mus musculus OX=10090 GN=Tenm1 PE=1 SV=1 |
| Q8VHD8\|HORN_MOUSE | Hornerin OS=Mus musculus OX=10090 GN=Hrnr PE=1 SV=1 |
| Q8BYG9\|EPHAA_MOUSE | Ephrin type-A receptor 10 OS=Mus musculus OX=10090 GN=Epha10 PE=2 SV=2 |
| Q8R2Z3\|S26A7_MOUSE | Anion exchange transporter OS=Mus musculus OX=10090 GN=Slc26a7 PE=2 SV=3 |
| Q8K4R9\|DLGP5_MOUSE | Disks large-associated protein 5 OS=Mus musculus OX=10090 GN=Dlgap5 PE=1 SV=2 |
| Q8BV57\|SRCRL_MOUSE | Soluble scavenger receptor cysteine-rich domain-containing protein SSC5D OS=Mus musculus OX=10090 GN=Ssc5d PE=1 SV=1 |
| P55850\|DSC3_MOUSE | Desmocollin-3 OS=Mus musculus OX=10090 GN=Dsc3 PE=1 SV=3 |
| P70302\|STIM1_MOUSE | Stromal interaction molecule 1 OS=Mus musculus OX=10090 GN=Stim1 PE=1 SV=2 |
| Q8BND5\|QSOX1_MOUSE | Sulfhydryl oxidase 1 OS=Mus musculus OX=10090 GN=Qsox1 PE=1 SV=1 |
| Q99K41\|EMIL1_MOUSE | EMILIN-1 OS=Mus musculus OX=10090 GN=Emilin1 PE=1 SV=1 |
| Q8R121\|ZPI_MOUSE | Protein Z-dependent protease inhibitor OS=Mus musculus OX=10090 GN=Serpina10 PE=1 SV=1 |
| Q5SX39\|MYH4_MOUSE | Myosin-4 OS=Mus musculus OX=10090 GN=Myh4 PE=1 SV=1 |
| P20357\|MTAP2_MOUSE | Microtubule-associated protein 2 OS=Mus musculus OX=10090 GN=Map2 PE=1 SV=2 |
| P19137\|LAMA1_MOUSE | Laminin subunit alpha-1 OS=Mus musculus OX=10090 GN=Lama1 PE=1 SV=2 |
| P12382\|PFKAL_MOUSE | ATP-dependent 6-phosphofructokinase, liver type OS=Mus musculus OX=10090 GN=Pfkl PE=1 SV=4 |
| O70138\|MMP8_MOUSE | Neutrophil collagenase OS=Mus musculus OX=10090 GN=Mmp8 PE=2 SV=2 |
| Q3UZZ4\|OLFM4_MOUSE | Olfactomedin-4 OS=Mus musculus OX=10090 GN=Olfm4 PE=2 SV=1 |
| Q6DFX2\|ANTR2_MOUSE | Anthrax toxin receptor 2 OS=Mus musculus OX=10090 GN=Antxr2 PE=1 SV=1 |
| P47810\|WEE1_MOUSE | Wee1-like protein kinase OS=Mus musculus OX=10090 GN=Wee1 PE=1 SV=2 |
| Q8BK48\|EST2E_MOUSE | Pyrethroid hydrolase Ces2e OS=Mus musculus OX=10090 GN=Ces2e PE=1 SV=1 |
| Q8CF98\|COL10_MOUSE | Collectin-10 OS=Mus musculus OX=10090 GN=Colec10 PE=2 SV=1 |
| Q9DC04\|RGS3_MOUSE | Regulator of G-protein signaling 3 OS=Mus musculus OX=10090 GN=Rgs3 PE=1 SV=2 |
| P05555\|ITAM_MOUSE | Integrin alpha-M OS=Mus musculus OX=10090 GN=Itgam PE=1 SV=2 |
| Q61909\|MTG8_MOUSE | Protein CBFA2T1 OS=Mus musculus OX=10090 GN=Runx1t1 PE=2 SV=1 |
| P12388\|PAI2_MOUSE | Plasminogen activator inhibitor 2, macrophage OS=Mus musculus OX=10090 GN=Serpinb2 PE=1 SV=1 |
| O09118\|NET1_MOUSE | Netrin-1 OS=Mus musculus OX=10090 GN=Ntn1 PE=1 SV=3 |
| P62192\|PRS4_MOUSE | 26S proteasome regulatory subunit 4 OS=Mus musculus OX=10090 GN=Psmc1 PE=1 SV=1 |
| P62334\|PRS10_MOUSE | 26S proteasome regulatory subunit 10B OS=Mus musculus OX=10090 GN=Psmc6 PE=1 SV=1 |
| Q5UAK0\|MIER1_MOUSE | Mesoderm induction early response protein 1 OS=Mus musculus OX=10090 GN=Mier1 PE=1 SV=2 |
| P80317\|TCPZ_MOUSE | T-complex protein 1 subunit zeta OS=Mus musculus OX=10090 GN=Cct6a PE=1 SV=3 |
| Q61035\|HARS1_MOUSE | Histidine--tRNA ligase, cytoplasmic OS=Mus musculus OX=10090 GN=Hars1 PE=1 SV=2 |
| Q8BMN3\|ACHB3_MOUSE | Neuronal acetylcholine receptor subunit beta-3 OS=Mus musculus OX=10090 GN=Chrnb3 PE=2 SV=1 |
| Q80W32\|DRC9_MOUSE | Dynein regulatory complex protein 9 OS=Mus musculus OX=10090 GN=Iqcg PE=1 SV=1 |
| Q9CPY7\|AMPL_MOUSE | Cytosol aminopeptidase OS=Mus musculus OX=10090 GN=Lap3 PE=1 SV=3 |
| P04186\|CFAB_MOUSE | Complement factor B OS=Mus musculus OX=10090 GN=Cfb PE=1 SV=2 |
| P48678\|LMNA_MOUSE | Prelamin-A/C OS=Mus musculus OX=10090 GN=Lmna PE=1 SV=2 |
| P09542\|MYL3_MOUSE | Myosin light chain 3 OS=Mus musculus OX=10090 GN=Myl3 PE=1 SV=4 |
| O89020\|AFAM_MOUSE | Afamin OS=Mus musculus OX=10090 GN=Afm PE=1 SV=2 |
| P97457\|MLRS_MOUSE | Myosin regulatory light chain 2, skeletal muscle isoform OS=Mus musculus OX=10090 GN=Mylpf PE=1 SV=3 |
| Q64314\|CD34_MOUSE | Hematopoietic progenitor cell antigen CD34 OS=Mus musculus OX=10090 GN=Cd34 PE=1 SV=1 |
| Q8K4L4\|POF1B_MOUSE | Protein POF1B OS=Mus musculus OX=10090 GN=Pof1b PE=2 SV=3 |
| Q91YP3\|DEOC_MOUSE | Deoxyribose-phosphate aldolase OS=Mus musculus OX=10090 GN=Dera PE=1 SV=1 |
| P58252\|EF2_MOUSE | Elongation factor 2 OS=Mus musculus OX=10090 GN=Eef2 PE=1 SV=2 |
| P63168\|DYL1_MOUSE | Dynein light chain 1, cytoplasmic OS=Mus musculus OX=10090 GN=Dynll1 PE=1 SV=1 |
| Q9D0M5\|DYL2_MOUSE | Dynein light chain 2, cytoplasmic OS=Mus musculus OX=10090 GN=Dynll2 PE=1 SV=1 |
| P12399\|CTL2A_MOUSE | Protein CTLA-2-alpha OS=Mus musculus OX=10090 GN=Ctla2a PE=2 SV=2 |
| P50543\|S10AB_MOUSE | Protein S100-A11 OS=Mus musculus OX=10090 GN=S100a11 PE=1 SV=1 |
| P04939\|MUP3_MOUSE | Major urinary protein 3 OS=Mus musculus OX=10090 GN=Mup3 PE=1 SV=1 |
| Q9Z0S4\|CLD13_MOUSE | Claudin-13 OS=Mus musculus OX=10090 GN=Cldn13 PE=1 SV=1 |
| O55135\|IF6_MOUSE | Eukaryotic translation initiation factor 6 OS=Mus musculus OX=10090 GN=Eif6 PE=1 SV=2 |
| Q5FW60\|MUP20_MOUSE | Major urinary protein 20 OS=Mus musculus OX=10090 GN=Mup20 PE=1 SV=1 |
| P18419\|SVS4_MOUSE | Seminal vesicle secretory protein 4 OS=Mus musculus OX=10090 GN=Svs4 PE=1 SV=2 |
| P30933\|SVS5_MOUSE | Seminal vesicle secretory protein 5 OS=Mus musculus OX=10090 GN=Svs5 PE=2 SV=1 |
| Q8R016\|BLMH_MOUSE | Bleomycin hydrolase OS=Mus musculus OX=10090 GN=Blmh PE=1 SV=1 |
| Q61805\|LBP_MOUSE | Lipopolysaccharide-binding protein OS=Mus musculus OX=10090 GN=Lbp PE=1 SV=2 |
