# Supplementary material for: Exercise Training Stimulates the Release of Glutathione Peroxidase 1 (GPX1)‐Enriched Extracellular Vesicles That Promote Angiogenesis
Source: FASEB J. 2026 Jun 18;40(12):e72052. doi: 10.1096/fj.202505096RR (PMC13278521; doi:10.1096/fj.202505096RR)
Supplement: Supplementary file 5 — Table S3: Proteins Shared Between SedVs and ExerVs. [file FSB2-40-e72052-s005.docx]

**Supplemental Table S3. Proteins Shared Between SedVs and ExerVs**

| **Accession** | **Description** |
| --- | --- |
| **Q60925\|DBP_MOUSE** | **D site-binding protein OS=Mus musculus OX=10090 GN=Dbp PE=1 SV=2** |
| **Q8C165\|P20D1_MOUSE** | **N-fatty-acyl-amino acid synthase/hydrolase PM20D1 OS=Mus musculus OX=10090 GN=Pm20d1 PE=1 SV=1** |
| **Q8K426\|RETNG_MOUSE** | **Resistin-like gamma OS=Mus musculus OX=10090 GN=Retnlg PE=1 SV=1** |
| **O70435\|PSA3_MOUSE** | **Proteasome subunit alpha type-3 OS=Mus musculus OX=10090 GN=Psma3 PE=1 SV=3** |
| **Q3SXB8\|COL11_MOUSE** | **Collectin-11 OS=Mus musculus OX=10090 GN=Colec11 PE=2 SV=2** |
| **P26041\|MOES_MOUSE** | **Moesin OS=Mus musculus OX=10090 GN=Msn PE=1 SV=3** |
| **P26043\|RADI_MOUSE** | **Radixin OS=Mus musculus OX=10090 GN=Rdx PE=1 SV=3** |
| **P26040\|EZRI_MOUSE** | **Ezrin OS=Mus musculus OX=10090 GN=Ezr PE=1 SV=3** |
| **Q80ZA4\|PKHL1_MOUSE** | **Fibrocystin-L OS=Mus musculus OX=10090 GN=Pkhd1l1 PE=1 SV=1** |
| **P07758\|A1AT1_MOUSE** | **Alpha-1-antitrypsin 1-1 OS=Mus musculus OX=10090 GN=Serpina1a PE=1 SV=4** |
| **P11276\|FINC_MOUSE** | **Fibronectin OS=Mus musculus OX=10090 GN=Fn1 PE=1 SV=4** |
| **Q6GQT1\|A2MG_MOUSE** | **Alpha-2-macroglobulin-P OS=Mus musculus OX=10090 GN=A2m PE=2 SV=2** |
| **Q9R1P3\|PSB2_MOUSE** | **Proteasome subunit beta type-2 OS=Mus musculus OX=10090 GN=Psmb2 PE=1 SV=1** |
| **Q63880\|EST3A_MOUSE** | **Carboxylesterase 3A OS=Mus musculus OX=10090 GN=Ces3a PE=1 SV=2** |
| **Q9ES30\|C1QT3_MOUSE** | **Complement C1q tumor necrosis factor-related protein 3 OS=Mus musculus OX=10090 GN=C1qtnf3 PE=2 SV=1** |
| **Q9R1P1\|PSB3_MOUSE** | **Proteasome subunit beta type-3 OS=Mus musculus OX=10090 GN=Psmb3 PE=1 SV=1** |
| **P22599\|A1AT2_MOUSE** | **Alpha-1-antitrypsin 1-2 OS=Mus musculus OX=10090 GN=Serpina1b PE=1 SV=2** |
| P08607\|C4BPA_MOUSE | C4b-binding protein OS=Mus musculus OX=10090 GN=C4bpa PE=1 SV=3 |
| P13020\|GELS_MOUSE | Gelsolin OS=Mus musculus OX=10090 GN=Gsn PE=1 SV=3 |
| Q00897\|A1AT4_MOUSE | Alpha-1-antitrypsin 1-4 OS=Mus musculus OX=10090 GN=Serpina1d PE=1 SV=1 |
| B5X0G2\|MUP17_MOUSE | Major urinary protein 17 OS=Mus musculus OX=10090 GN=Mup17 PE=2 SV=2 |
| Q80YX1\|TENA_MOUSE | Tenascin OS=Mus musculus OX=10090 GN=Tnc PE=1 SV=1 |
| Q02257\|PLAK_MOUSE | Junction plakoglobin OS=Mus musculus OX=10090 GN=Jup PE=1 SV=3 |
| O08677\|KNG1_MOUSE | Kininogen-1 OS=Mus musculus OX=10090 GN=Kng1 PE=1 SV=1 |
| Q8BH61\|F13A_MOUSE | Coagulation factor XIII A chain OS=Mus musculus OX=10090 GN=F13a1 PE=1 SV=3 |
| Q61171\|PRDX2_MOUSE | Peroxiredoxin-2 OS=Mus musculus OX=10090 GN=Prdx2 PE=1 SV=3 |
| Q9R1P0\|PSA4_MOUSE | Proteasome subunit alpha type-4 OS=Mus musculus OX=10090 GN=Psma4 PE=1 SV=1 |
| Q32M00\|CRBL2_MOUSE | cAMP-responsive element-binding protein-like 2 OS=Mus musculus OX=10090 GN=Crebl2 PE=1 SV=1 |
| P28665\|MUG1_MOUSE | Murinoglobulin-1 OS=Mus musculus OX=10090 GN=Mug1 PE=1 SV=3 |
| Q61703\|ITIH2_MOUSE | Inter-alpha-trypsin inhibitor heavy chain H2 OS=Mus musculus OX=10090 GN=Itih2 PE=1 SV=1 |
| Q0VEE6\|ZN800_MOUSE | Zinc finger protein 800 OS=Mus musculus OX=10090 GN=Znf800 PE=1 SV=1 |
| P02089\|HBB2_MOUSE | Hemoglobin subunit beta-2 OS=Mus musculus OX=10090 GN=Hbb-b2 PE=1 SV=2 |
| Q8K0E8\|FIBB_MOUSE | Fibrinogen beta chain OS=Mus musculus OX=10090 GN=Fgb PE=1 SV=1 |
| Q68FD5\|CLH1_MOUSE | Clathrin heavy chain 1 OS=Mus musculus OX=10090 GN=Cltc PE=1 SV=3 |
| Q60994\|ADIPO_MOUSE | Adiponectin OS=Mus musculus OX=10090 GN=Adipoq PE=1 SV=2 |
| Q61702\|ITIH1_MOUSE | Inter-alpha-trypsin inhibitor heavy chain H1 OS=Mus musculus OX=10090 GN=Itih1 PE=1 SV=2 |
| D3YYU8\|OBSL1_MOUSE | Obscurin-like protein 1 OS=Mus musculus OX=10090 GN=Obsl1 PE=1 SV=1 |
| P13609\|SRGN_MOUSE | Serglycin OS=Mus musculus OX=10090 GN=Srgn PE=1 SV=1 |
| P08032\|SPTA1_MOUSE | Spectrin alpha chain, erythrocytic 1 OS=Mus musculus OX=10090 GN=Spta1 PE=1 SV=3 |
| P17742\|PPIA_MOUSE | Peptidyl-prolyl cis-trans isomerase A OS=Mus musculus OX=10090 GN=Ppia PE=1 SV=2 |
| P48759\|PTX3_MOUSE | Pentraxin-related protein PTX3 OS=Mus musculus OX=10090 GN=Ptx3 PE=1 SV=2 |
| Q00898\|A1AT5_MOUSE | Alpha-1-antitrypsin 1-5 OS=Mus musculus OX=10090 GN=Serpina1e PE=1 SV=1 |
| Q02013\|AQP1_MOUSE | Aquaporin-1 OS=Mus musculus OX=10090 GN=Aqp1 PE=1 SV=3 |
| Q8VCM7\|FIBG_MOUSE | Fibrinogen gamma chain OS=Mus musculus OX=10090 GN=Fgg PE=1 SV=1 |
| Q8K182\|CO8A_MOUSE | Complement component C8 alpha chain OS=Mus musculus OX=10090 GN=C8a PE=1 SV=1 |
| Q96DY5\|RN112_MOUSE | RING finger protein 112 OS=Mus musculus OX=10090 GN=Rnf112 PE=1 SV=1 |
| P29788\|VTNC_MOUSE | Vitronectin OS=Mus musculus OX=10090 GN=Vtn PE=1 SV=2 |
| P01027\|CO3_MOUSE | Complement C3 OS=Mus musculus OX=10090 GN=C3 PE=1 SV=3 |
| P16858\|G3P_MOUSE | Glyceraldehyde-3-phosphate dehydrogenase OS=Mus musculus OX=10090 GN=Gapdh PE=1 SV=2 |
| P08228\|SODC_MOUSE | Superoxide dismutase [Cu-Zn] OS=Mus musculus OX=10090 GN=Sod1 PE=1 SV=2 |
| P62737\|ACTA_MOUSE | Actin, aortic smooth muscle OS=Mus musculus OX=10090 GN=Acta2 PE=1 SV=1 |
| P68134\|ACTS_MOUSE | Actin, alpha skeletal muscle OS=Mus musculus OX=10090 GN=Acta1 PE=1 SV=1 |
| P68033\|ACTC_MOUSE | Actin, alpha cardiac muscle 1 OS=Mus musculus OX=10090 GN=Actc1 PE=1 SV=1 |
| P63268\|ACTH_MOUSE | Actin, gamma-enteric smooth muscle OS=Mus musculus OX=10090 GN=Actg2 PE=1 SV=1 |
| P04919\|B3AT_MOUSE | Band 3 anion transport protein OS=Mus musculus OX=10090 GN=Slc4a1 PE=1 SV=1 |
| P39039\|MBL1_MOUSE | Mannose-binding protein A OS=Mus musculus OX=10090 GN=Mbl1 PE=1 SV=1 |
| Q01279\|EGFR_MOUSE | Epidermal growth factor receptor OS=Mus musculus OX=10090 GN=Egfr PE=1 SV=1 |
| Q3UZ09\|C1RL_MOUSE | Complement C1r subcomponent-like protein OS=Mus musculus OX=10090 GN=C1rl PE=1 SV=1 |
| Q9ESB3\|HRG_MOUSE | Histidine-rich glycoprotein OS=Mus musculus OX=10090 GN=Hrg PE=1 SV=2 |
| O35930\|GP1BA_MOUSE | Platelet glycoprotein Ib alpha chain OS=Mus musculus OX=10090 GN=Gp1ba PE=1 SV=2 |
| Q02105\|C1QC_MOUSE | Complement C1q subcomponent subunit C OS=Mus musculus OX=10090 GN=C1qc PE=1 SV=2 |
| Q60692\|PSB6_MOUSE | Proteasome subunit beta type-6 OS=Mus musculus OX=10090 GN=Psmb6 PE=1 SV=3 |
| P28798\|GRN_MOUSE | Progranulin OS=Mus musculus OX=10090 GN=Grn PE=1 SV=2 |
| P01942\|HBA_MOUSE | Hemoglobin subunit alpha OS=Mus musculus OX=10090 GN=Hba PE=1 SV=2 |
| Q61704\|ITIH3_MOUSE | Inter-alpha-trypsin inhibitor heavy chain H3 OS=Mus musculus OX=10090 GN=Itih3 PE=1 SV=3 |
| Q8R3G9\|TSN8_MOUSE | Tetraspanin-8 OS=Mus musculus OX=10090 GN=Tspan8 PE=1 SV=1 |
| P10493\|NID1_MOUSE | Nidogen-1 OS=Mus musculus OX=10090 GN=Nid1 PE=1 SV=2 |
| P46412\|GPX3_MOUSE | Glutathione peroxidase 3 OS=Mus musculus OX=10090 GN=Gpx3 PE=1 SV=2 |
| Q61247\|A2AP_MOUSE | Alpha-2-antiplasmin OS=Mus musculus OX=10090 GN=Serpinf2 PE=1 SV=1 |
| Q07968\|F13B_MOUSE | Coagulation factor XIII B chain OS=Mus musculus OX=10090 GN=F13b PE=1 SV=2 |
| P49722\|PSA2_MOUSE | Proteasome subunit alpha type-2 OS=Mus musculus OX=10090 GN=Psma2 PE=1 SV=3 |
| P01029\|CO4B_MOUSE | Complement C4-B OS=Mus musculus OX=10090 GN=C4b PE=1 SV=3 |
| P05064\|ALDOA_MOUSE | Fructose-bisphosphate aldolase A OS=Mus musculus OX=10090 GN=Aldoa PE=1 SV=2 |
| O35955\|PSB10_MOUSE | Proteasome subunit beta type-10 OS=Mus musculus OX=10090 GN=Psmb10 PE=1 SV=1 |
| P60710\|ACTB_MOUSE | Actin, cytoplasmic 1 OS=Mus musculus OX=10090 GN=Actb PE=1 SV=1 |
| P63260\|ACTG_MOUSE | Actin, cytoplasmic 2 OS=Mus musculus OX=10090 GN=Actg1 PE=1 SV=1 |
| O88491\|NSD1_MOUSE | Histone-lysine N-methyltransferase, H3 lysine-36 specific OS=Mus musculus OX=10090 GN=Nsd1 PE=1 SV=1 |
| P20029\|BIP_MOUSE | Endoplasmic reticulum chaperone BiP OS=Mus musculus OX=10090 GN=Hspa5 PE=1 SV=3 |
| P48193\|EPB41_MOUSE | Protein 4.1 OS=Mus musculus OX=10090 GN=Epb41 PE=1 SV=2 |
| P20918\|PLMN_MOUSE | Plasminogen OS=Mus musculus OX=10090 GN=Plg PE=1 SV=3 |
| P52430\|PON1_MOUSE | Serum paraoxonase/arylesterase 1 OS=Mus musculus OX=10090 GN=Pon1 PE=1 SV=2 |
| P02104\|HBE_MOUSE | Hemoglobin subunit epsilon-Y2 OS=Mus musculus OX=10090 GN=Hbb-y PE=1 SV=2 |
| Q8CIZ8\|VWF_MOUSE | von Willebrand factor OS=Mus musculus OX=10090 GN=Vwf PE=1 SV=2 |
| P54116\|STOM_MOUSE | Stomatin OS=Mus musculus OX=10090 GN=Stom PE=1 SV=3 |
| P31532\|SAA4_MOUSE | Serum amyloid A-4 protein OS=Mus musculus OX=10090 GN=Saa4 PE=1 SV=2 |
| Q03311\|CHLE_MOUSE | Cholinesterase OS=Mus musculus OX=10090 GN=Bche PE=1 SV=2 |
| Q01339\|APOH_MOUSE | Beta-2-glycoprotein 1 OS=Mus musculus OX=10090 GN=Apoh PE=1 SV=1 |
| Q9CWH6\|PSMA8_MOUSE | Proteasome subunit alpha type-8 OS=Mus musculus OX=10090 GN=Psma8 PE=1 SV=1 |
| P07759\|SPA3K_MOUSE | Serine protease inhibitor A3K OS=Mus musculus OX=10090 GN=Serpina3k PE=1 SV=2 |
| Q91WP0\|MASP2_MOUSE | Mannan-binding lectin serine protease 2 OS=Mus musculus OX=10090 GN=Masp2 PE=1 SV=1 |
| P06909\|CFAH_MOUSE | Complement factor H OS=Mus musculus OX=10090 GN=Cfh PE=1 SV=2 |
| P26039\|TLN1_MOUSE | Talin-1 OS=Mus musculus OX=10090 GN=Tln1 PE=1 SV=2 |
| Q61838\|PZP_MOUSE | Pregnancy zone protein OS=Mus musculus OX=10090 GN=Pzp PE=1 SV=3 |
| O70165\|FCN1_MOUSE | Ficolin-1 OS=Mus musculus OX=10090 GN=Fcn1 PE=1 SV=1 |
| Q9DBB9\|CPN2_MOUSE | Carboxypeptidase N subunit 2 OS=Mus musculus OX=10090 GN=Cpn2 PE=1 SV=2 |
| P55065\|PLTP_MOUSE | Phospholipid transfer protein OS=Mus musculus OX=10090 GN=Pltp PE=1 SV=1 |
| E9Q557\|DESP_MOUSE | Desmoplakin OS=Mus musculus OX=10090 GN=Dsp PE=1 SV=1 |
| P07309\|TTHY_MOUSE | Transthyretin OS=Mus musculus OX=10090 GN=Ttr PE=1 SV=1 |
| P14106\|C1QB_MOUSE | Complement C1q subcomponent subunit B OS=Mus musculus OX=10090 GN=C1qb PE=1 SV=2 |
| Q9QUM0\|ITA2B_MOUSE | Integrin alpha-IIb OS=Mus musculus OX=10090 GN=Itga2b PE=1 SV=2 |
| P11152\|LIPL_MOUSE | Lipoprotein lipase OS=Mus musculus OX=10090 GN=Lpl PE=1 SV=3 |
| P98064\|MASP1_MOUSE | Mannan-binding lectin serine protease 1 OS=Mus musculus OX=10090 GN=Masp1 PE=1 SV=2 |
| P99026\|PSB4_MOUSE | Proteasome subunit beta type-4 OS=Mus musculus OX=10090 GN=Psmb4 PE=1 SV=1 |
| Q02357\|ANK1_MOUSE | Ankyrin-1 OS=Mus musculus OX=10090 GN=Ank1 PE=1 SV=2 |
| Q07797\|LG3BP_MOUSE | Galectin-3-binding protein OS=Mus musculus OX=10090 GN=Lgals3bp PE=1 SV=1 |
| Q9Z2U1\|PSA5_MOUSE | Proteasome subunit alpha type-5 OS=Mus musculus OX=10090 GN=Psma5 PE=1 SV=1 |
| O35516\|NOTC2_MOUSE | Neurogenic locus notch homolog protein 2 OS=Mus musculus OX=10090 GN=Notch2 PE=1 SV=2 |
| Q6X893\|CTL1_MOUSE | Choline transporter-like protein 1 OS=Mus musculus OX=10090 GN=Slc44a1 PE=1 SV=3 |
| P98086\|C1QA_MOUSE | Complement C1q subcomponent subunit A OS=Mus musculus OX=10090 GN=C1qa PE=1 SV=2 |
| O88783\|FA5_MOUSE | Coagulation factor V OS=Mus musculus OX=10090 GN=F5 PE=1 SV=1 |
| Q08879\|FBLN1_MOUSE | Fibulin-1 OS=Mus musculus OX=10090 GN=Fbln1 PE=1 SV=2 |
| O70570\|PIGR_MOUSE | Polymeric immunoglobulin receptor OS=Mus musculus OX=10090 GN=Pigr PE=1 SV=1 |
| Q9Z2U0\|PSA7_MOUSE | Proteasome subunit alpha type-7 OS=Mus musculus OX=10090 GN=Psma7 PE=1 SV=1 |
| P17156\|HSP72_MOUSE | Heat shock-related 70 kDa protein 2 OS=Mus musculus OX=10090 GN=Hspa2 PE=1 SV=2 |
| Q8CG16\|C1RA_MOUSE | Complement C1r-A subcomponent OS=Mus musculus OX=10090 GN=C1ra PE=1 SV=1 |
| P02468\|LAMC1_MOUSE | Laminin subunit gamma-1 OS=Mus musculus OX=10090 GN=Lamc1 PE=1 SV=2 |
| P11680\|PROP_MOUSE | Properdin OS=Mus musculus OX=10090 GN=Cfp PE=1 SV=2 |
| P35441\|TSP1_MOUSE | Thrombospondin-1 OS=Mus musculus OX=10090 GN=Thbs1 PE=1 SV=1 |
| P29699\|FETUA_MOUSE | Alpha-2-HS-glycoprotein OS=Mus musculus OX=10090 GN=Ahsg PE=1 SV=1 |
| Q8BXK8\|AGAP1_MOUSE | Arf-GAP with GTPase, ANK repeat and PH domain-containing protein 1 OS=Mus musculus OX=10090 GN=Agap1 PE=1 SV=1 |
| Q01853\|TERA_MOUSE | Transitional endoplasmic reticulum ATPase OS=Mus musculus OX=10090 GN=Vcp PE=1 SV=4 |
| Q64514\|TPP2_MOUSE | Tripeptidyl-peptidase 2 OS=Mus musculus OX=10090 GN=Tpp2 PE=1 SV=3 |
| P97290\|IC1_MOUSE | Plasma protease C1 inhibitor OS=Mus musculus OX=10090 GN=Serping1 PE=1 SV=3 |
| P41317\|MBL2_MOUSE | Mannose-binding protein C OS=Mus musculus OX=10090 GN=Mbl2 PE=1 SV=2 |
| Q8CFG8\|CS1B_MOUSE | Complement C1s-B subcomponent OS=Mus musculus OX=10090 GN=C1sb PE=2 SV=1 |
| Q8CG14\|CS1A_MOUSE | Complement C1s-A subcomponent OS=Mus musculus OX=10090 GN=C1sa PE=2 SV=2 |
| Q921I1\|TRFE_MOUSE | Serotransferrin OS=Mus musculus OX=10090 GN=Tf PE=1 SV=1 |
| O70362\|PHLD_MOUSE | Phosphatidylinositol-glycan-specific phospholipase D OS=Mus musculus OX=10090 GN=Gpld1 PE=1 SV=1 |
| Q8CFG9\|C1RB_MOUSE | Complement C1r-B subcomponent OS=Mus musculus OX=10090 GN=C1rb PE=2 SV=1 |
| P49222\|EPB42_MOUSE | Protein 4.2 OS=Mus musculus OX=10090 GN=Epb42 PE=1 SV=3 |
| P03995\|GFAP_MOUSE | Glial fibrillary acidic protein OS=Mus musculus OX=10090 GN=Gfap PE=1 SV=4 |
| P00920\|CAH2_MOUSE | Carbonic anhydrase 2 OS=Mus musculus OX=10090 GN=Ca2 PE=1 SV=4 |
| P63017\|HSP7C_MOUSE | Heat shock cognate 71 kDa protein OS=Mus musculus OX=10090 GN=Hspa8 PE=1 SV=1 |
| P08752\|GNAI2_MOUSE | Guanine nucleotide-binding protein G(i) subunit alpha-2 OS=Mus musculus OX=10090 GN=Gnai2 PE=1 SV=5 |
| O09061\|PSB1_MOUSE | Proteasome subunit beta type-1 OS=Mus musculus OX=10090 GN=Psmb1 PE=1 SV=1 |
| E9PV24\|FIBA_MOUSE | Fibrinogen alpha chain OS=Mus musculus OX=10090 GN=Fga PE=1 SV=1 |
| Q8K0U4\|HS12A_MOUSE | Heat shock 70 kDa protein 12A OS=Mus musculus OX=10090 GN=Hspa12a PE=1 SV=1 |
| P32261\|ANT3_MOUSE | Antithrombin-III OS=Mus musculus OX=10090 GN=Serpinc1 PE=1 SV=1 |
| O08992\|SDCB1_MOUSE | Syntenin-1 OS=Mus musculus OX=10090 GN=Sdcbp PE=1 SV=1 |
| P01898\|HA10_MOUSE | H-2 class I histocompatibility antigen, Q10 alpha chain OS=Mus musculus OX=10090 GN=H2-Q10 PE=1 SV=3 |
| Q07456\|AMBP_MOUSE | Protein AMBP OS=Mus musculus OX=10090 GN=Ambp PE=1 SV=2 |
| P06684\|CO5_MOUSE | Complement C5 OS=Mus musculus OX=10090 GN=C5 PE=1 SV=2 |
| Q03734\|SPA3M_MOUSE | Serine protease inhibitor A3M OS=Mus musculus OX=10090 GN=Serpina3m PE=1 SV=2 |
| P70195\|PSB7_MOUSE | Proteasome subunit beta type-7 OS=Mus musculus OX=10090 GN=Psmb7 PE=1 SV=1 |
| Q8BH35\|CO8B_MOUSE | Complement component C8 beta chain OS=Mus musculus OX=10090 GN=C8b PE=1 SV=1 |
| P42703\|LIFR_MOUSE | Leukemia inhibitory factor receptor OS=Mus musculus OX=10090 GN=Lifr PE=1 SV=1 |
| Q60841\|RELN_MOUSE | Reelin OS=Mus musculus OX=10090 GN=Reln PE=1 SV=3 |
| Q8VCG4\|CO8G_MOUSE | Complement component C8 gamma chain OS=Mus musculus OX=10090 GN=C8g PE=1 SV=1 |
| Q8BWQ1\|UD2A3_MOUSE | UDP-glucuronosyltransferase 2A3 OS=Mus musculus OX=10090 GN=Ugt2a3 PE=1 SV=1 |
| Q01768\|NDKB_MOUSE | Nucleoside diphosphate kinase B OS=Mus musculus OX=10090 GN=Nme2 PE=1 SV=1 |
| P15532\|NDKA_MOUSE | Nucleoside diphosphate kinase A OS=Mus musculus OX=10090 GN=Nme1 PE=1 SV=1 |
| Q8BHN3\|GANAB_MOUSE | Neutral alpha-glucosidase AB OS=Mus musculus OX=10090 GN=Ganab PE=1 SV=1 |
| Q91WP6\|SPA3N_MOUSE | Serine protease inhibitor A3N OS=Mus musculus OX=10090 GN=Serpina3n PE=1 SV=1 |
| Q9JJN5\|CBPN_MOUSE | Carboxypeptidase N catalytic chain OS=Mus musculus OX=10090 GN=Cpn1 PE=1 SV=1 |
| Q8C015\|PAK5_MOUSE | Serine/threonine-protein kinase PAK 5 OS=Mus musculus OX=10090 GN=Pak5 PE=1 SV=1 |
| P02469\|LAMB1_MOUSE | Laminin subunit beta-1 OS=Mus musculus OX=10090 GN=Lamb1 PE=1 SV=3 |
| Q9Z1T2\|TSP4_MOUSE | Thrombospondin-4 OS=Mus musculus OX=10090 GN=Thbs4 PE=1 SV=1 |
| Q9QUM9\|PSA6_MOUSE | Proteasome subunit alpha type-6 OS=Mus musculus OX=10090 GN=Psma6 PE=1 SV=1 |
| P53986\|MOT1_MOUSE | Monocarboxylate transporter 1 OS=Mus musculus OX=10090 GN=Slc16a1 PE=1 SV=1 |
| P23953\|EST1C_MOUSE | Carboxylesterase 1C OS=Mus musculus OX=10090 GN=Ces1c PE=1 SV=4 |
| Q8R0Z6\|ANGL6_MOUSE | Angiopoietin-related protein 6 OS=Mus musculus OX=10090 GN=Angptl6 PE=2 SV=1 |
| Q60675\|LAMA2_MOUSE | Laminin subunit alpha-2 OS=Mus musculus OX=10090 GN=Lama2 PE=1 SV=2 |
| P02088\|HBB1_MOUSE | Hemoglobin subunit beta-1 OS=Mus musculus OX=10090 GN=Hbb-b1 PE=1 SV=2 |
| Q61129\|CFAI_MOUSE | Complement factor I OS=Mus musculus OX=10090 GN=Cfi PE=1 SV=3 |
| Q9R0M4\|PODXL_MOUSE | Podocalyxin OS=Mus musculus OX=10090 GN=Podxl PE=1 SV=2 |
| P21614\|VTDB_MOUSE | Vitamin D-binding protein OS=Mus musculus OX=10090 GN=Gc PE=1 SV=2 |
| O54890\|ITB3_MOUSE | Integrin beta-3 OS=Mus musculus OX=10090 GN=Itgb3 PE=1 SV=2 |
| O55234\|PSB5_MOUSE | Proteasome subunit beta type-5 OS=Mus musculus OX=10090 GN=Psmb5 PE=1 SV=3 |
| P40240\|CD9_MOUSE | CD9 antigen OS=Mus musculus OX=10090 GN=Cd9 PE=1 SV=2 |
| Q9R1P4\|PSA1_MOUSE | Proteasome subunit alpha type-1 OS=Mus musculus OX=10090 GN=Psma1 PE=1 SV=1 |
| Q06890\|CLUS_MOUSE | Clusterin OS=Mus musculus OX=10090 GN=Clu PE=1 SV=1 |
| P11087\|CO1A1_MOUSE | Collagen alpha-1(I) chain OS=Mus musculus OX=10090 GN=Col1a1 PE=1 SV=4 |
| Q8C0D5\|EFL1_MOUSE | Elongation factor-like GTPase 1 OS=Mus musculus OX=10090 GN=Efl1 PE=1 SV=1 |
| Q91ZX7\|LRP1_MOUSE | Prolow-density lipoprotein receptor-related protein 1 OS=Mus musculus OX=10090 GN=Lrp1 PE=1 SV=1 |
| Q9QWK4\|CD5L_MOUSE | CD5 antigen-like OS=Mus musculus OX=10090 GN=Cd5l PE=1 SV=3 |
| Q61735\|CD47_MOUSE | Leukocyte surface antigen CD47 OS=Mus musculus OX=10090 GN=Cd47 PE=1 SV=2 |
| Q8BPB5\|FBLN3_MOUSE | EGF-containing fibulin-like extracellular matrix protein 1 OS=Mus musculus OX=10090 GN=Efemp1 PE=1 SV=1 |

**Bold = P<0.05**
